# A genetically encoded Ca^2+^ indicator based on circularly permutated sea anemone red fluorescent protein eqFP578

**DOI:** 10.1186/s12915-018-0480-0

**Published:** 2018-01-16

**Authors:** Yi Shen, Hod Dana, Ahmed S. Abdelfattah, Ronak Patel, Jamien Shea, Rosana S. Molina, Bijal Rawal, Vladimir Rancic, Yu-Fen Chang, Lanshi Wu, Yingche Chen, Yong Qian, Matthew D. Wiens, Nathan Hambleton, Klaus Ballanyi, Thomas E. Hughes, Mikhail Drobizhev, Douglas S. Kim, Minoru Koyama, Eric R. Schreiter, Robert E. Campbell

**Affiliations:** 1 Department of Chemistry, University of Alberta, Edmonton, Alberta T6G 2G2 Canada; 2 Janelia Research Campus, Howard Hughes Medical Institute, Ashburn, VA 20147 USA; 3 Department of Cell Biology and Neuroscience, Montana State University, Bozeman, MT 59717 USA; 4 Department of Physiology, University of Alberta, Edmonton, Alberta T6G 2H7 Canada; 5 LumiSTAR Biotechnology Incorporation, Nangang District, Taipei City, 115 Taiwan; 6 Present address: Department of Neurosciences, Lerner Research Institute, Cleveland Clinic Foundation, Cleveland, OH 4195 USA; 7 Present address: Janelia Research Campus, Howard Hughes Medical Institute, Ashburn, VA 20147 USA

## Abstract

**Background:**

Genetically encoded calcium ion (Ca^2+^) indicators (GECIs) are indispensable tools for measuring Ca^2+^ dynamics and neuronal activities in vitro and in vivo. Red fluorescent protein (RFP)-based GECIs have inherent advantages relative to green fluorescent protein-based GECIs due to the longer wavelength light used for excitation. Longer wavelength light is associated with decreased phototoxicity and deeper penetration through tissue. Red GECI can also enable multicolor visualization with blue- or cyan-excitable fluorophores.

**Results:**

Here we report the development, structure, and validation of a new RFP-based GECI, K-GECO1, based on a circularly permutated RFP derived from the sea anemone *Entacmaea quadricolor*. We have characterized the performance of K-GECO1 in cultured HeLa cells, dissociated neurons, stem-cell-derived cardiomyocytes, organotypic brain slices, zebrafish spinal cord in vivo, and mouse brain in vivo.

**Conclusion:**

K-GECO1 is the archetype of a new lineage of GECIs based on the RFP eqFP578 scaffold. It offers high sensitivity and fast kinetics, similar or better than those of current state-of-the-art indicators, with diminished lysosomal accumulation and minimal blue-light photoactivation. Further refinements of the K-GECO1 lineage could lead to further improved variants with overall performance that exceeds that of the most highly optimized red GECIs.

**Electronic supplementary material:**

The online version of this article (doi:10.1186/s12915-018-0480-0) contains supplementary material, which is available to authorized users.

## Background

**Fig. 1:**
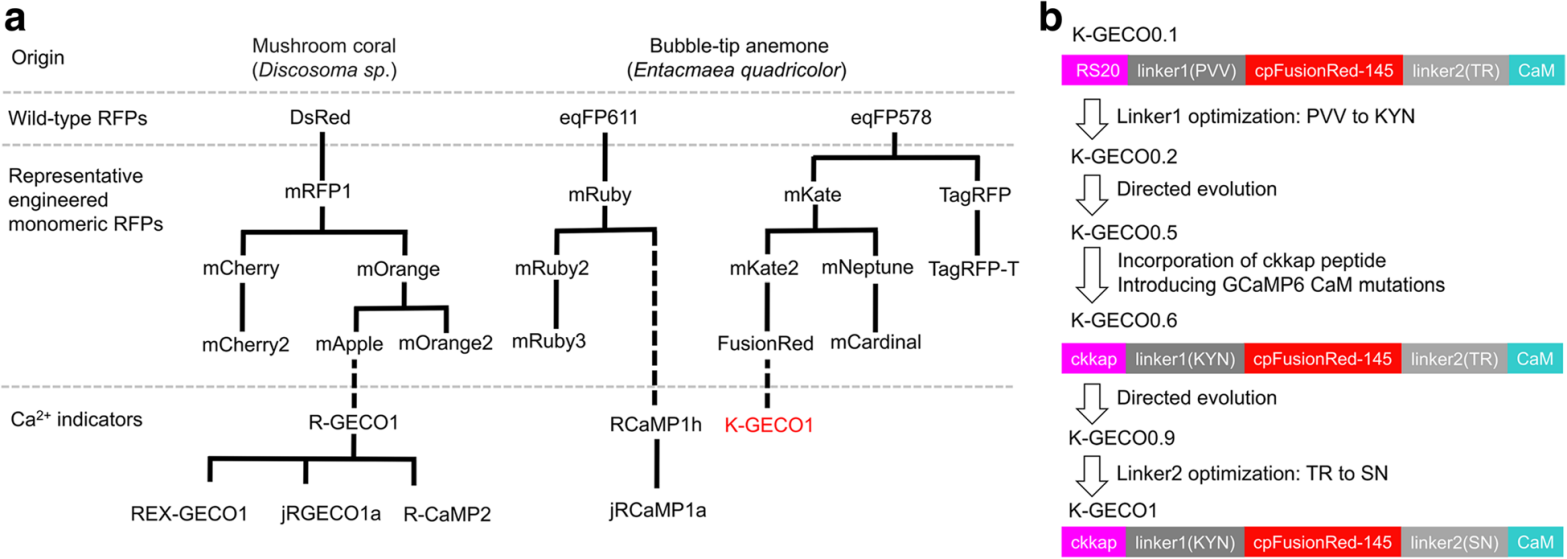
Design and development of K-GECO1. **a** Selected RFP and RFP-based Ca^2+^ indicator genealogy. **b** Schematic illustration of K-GECO1 design and engineering. RFP red fluorescent protein

Protein engineering efforts have yielded three major lineages of monomeric red fluorescent proteins (RFPs) derived from their naturally oligomeric precursors (Fig. 1a). One lineage comes from the *Discosoma* sp. mushroom coral RFP, DsRed, and includes the first monomeric RFP, mRFP1 [1], and the mRFP1-derived mFruit variants such as mCherry, mCherry2, mOrange, and mApple [2–4]. The second and third lineages stem from the sea anemone *Entacmaea quadricolor* RFPs eqFP578 [5] and eqFP611 [6], respectively. EqFP578 is the progenitor of the bright monomeric proteins TagRFP, TagRFP-T, mKate, mKate2, and the low-cytotoxicity variant FusionRed [5, 7–9]. Engineering of eqFP611 produced mRuby, mRuby2, and mRuby3, a line of RFPs with relatively large Stokes shift and bright red fluorescence [10–12]. Together, these three lineages of monomeric RFPs are commonly used in a variety of fluorescence imaging applications and have served as templates for developing red fluorescent indicators of various biochemical activities [13].

Among the many fluorescent-protein-based indicators of biochemical activity, genetically encoded calcium ion (Ca^2+^) indicators (GECIs) are particularly versatile tools. Most notably, they enable imaging of neuronal activity in contexts ranging from dissociated neurons in vitro to brain activity in behaving animals [14]. Green fluorescent GCaMPs, in particular, have proven extremely useful for imaging Ca^2+^ activities in various neural systems [15–17]. The development of the first single RFP-based Ca^2+^ indicators, the DsRed-derived R-GECO1 [18] and eqFP611-derived RCaMP1h [19], unlocked new opportunities for simultaneous multicolor optical imaging. Further engineering of R-GECO1 produced a number of improved and altered variants, including R-CaMP1.07, R-GECO1.2, CAR-GECO1, O-GECO1, R-CaMP2, and REX-GECO1 [20–23]. Optimization of R-GECO1 and RCaMP1h for detection of neuronal action potentials produced jRGECO1a, jRCaMP1a, and jRCaMP1b [24]. One limitation of the R-GECO series of GECIs is that they inherited undesirable blue-light-activated photoswitching behavior that was also present in the DsRed-derived template (mApple) from which they were engineered [3, 19, 25, 26]. Accordingly, when combining the R-GECO series of Ca^2+^ indicators with optogenetic actuators, extra care must be taken to differentiate true responses from artifacts caused by photoactivation [19, 21]. RCaMP variants do not show photoswitching under blue illumination but they are less responsive than R-GECO variants in terms of fluorescence change upon Ca^2+^ binding [19, 24]. Like many DsRed-derived RFPs, R-GECO variants have a propensity to accumulate in lysosomes and form brightly fluorescent (but non-functional) puncta during long-term neuronal expression [27–29]. These puncta can complicate image analysis and may compromise long-term cell viability. Notably, transgenic mice expressing RCaMP1.07 (equivalent to R-GECO1 K47V, T49V with a C-terminal peptide extension) exhibit stable and widespread neuronal expression, despite the formation of numerous puncta [30].

The drawbacks associated with the DsRed- and eqFP611-derived GECIs motivated us to explore a new RFP template for development of red GECIs. As mentioned above, some DsRed-derived RFPs, such as mOrange and mCherry, have been reported to exhibit relatively dim fluorescence and/or puncta formation, when transgenically expressed in mice brains [31]. In contrast, eqFP578-derived RFPs TagRFP-T and mKate2 have been reported to exhibit bright fluorescence without puncta formation in vivo [31]. The eqFP611-derived mRuby has been reported to have the highest cytotoxicity among various RFPs [9]. Based on these literature reports, and reinforced by observations in our own lab, we reasoned that using an eqFP578-derived RFP as a template for the development of a new red GECI could potentially address the limitations of R-GECO, and possibly offer better performance in vivo. Here we report our efforts to design, engineer, characterize, and validate a new red GECI, K-GECO1, based on the eqFP578 variant FusionRed [9].

## Results

### Design and engineering of K-GECO1

We initially selected two eqFP578-derived RFPs, mKate2 [8] and its low-cytotoxicity variant FusionRed [9], as templates to construct a red Ca^2+^ indicator. Both mKate2 and FusionRed scaffolds were circularly permutated (cp) at residue Ser143 (numbering according to mKate crystal structure [32], PDB: 3BXB), which is the same permutation site used in GCaMPs and R-GECOs [18, 33]. Both cpRFPs were genetically inserted between N-terminal chicken myosin light-chain kinase peptide RS20 and C-terminal calmodulin (CaM) from R-GECO1. The resulting indicator prototype based on the cpmKate2 scaffold was not fluorescent, in accordance with a previous study of mKate circular permutation [34], and therefore, no further optimization was pursued. In contrast, the cpFusionRed-based design (designated K-GECO0.1) (Fig. 1b), was dimly fluorescent when expressed in *Escherichia coli* colonies for 48 h at room temperature. The extracted protein showed a 20% fluorescence emission intensity increase upon addition of Ca^2+^. To improve the function of this prototype indicator further, we first performed random mutagenesis of the peptide linker between the RS20 peptide and cpFusionRed (linker1), which is Pro30-Val31-Val32 as in R-GECO1 (numbered as in Additional file 1: Figure S1). Screening of this targeted mutagenesis library led to identification of the Pro30Lys-Val31Tyr-Val32Asn variant with visible red fluorescence in *E. coli* after overnight incubation. This variant, termed K-GECO0.2, exhibited a twofold fluorescence emission intensity increase upon Ca^2+^ binding. K-GECO0.2 was subjected to further directed protein evolution for brightness and to increase the Ca^2+^-induced fluorescence intensity change. In each round of directed evolution, error-prone polymerase chain reaction (EP-PCR) was used to create a variant library. After visual inspection of the plated library, the brightest fluorescent colonies were picked, cultured, and the protein purified and tested for its Ca^2+^ response. The pool of variants with the largest Ca^2+^-dependent fluorescence changes served as templates for the next round of evolution. After three rounds, an improved variant K-GECO0.5 was produced. Initial characterization of K-GECO0.5 indicated a relatively low Ca^2+^ affinity with a *K*_d_ close to 1 μM. To overcome this limitation, we engineered K-GECO0.6 using an approach similar to the one used by Inoue et al. to develop R-CaMP2 [23]. Following the strategy of Inoue et al., we incorporated the rat CaM-dependent kinase kinase peptide (ckkap) in place of RS20, and introduced GCaMP6 mutations Asn342Ser, Asp343Tyr, Thr344Arg, and Ser346Thr into the CaM domain [23]. An additional three rounds of directed evolution led to K-GECO0.9. In the final step of engineering, we performed saturation mutagenesis of the linker between cpFusionRed and CaM (linker2). Screening of the library identified a variant with linker2 changed from Thr265-Arg266 into Ser265-Asn266. This final variant was designated as K-GECO1 (Fig. 1b).

### In vitro characterization of K-GECO1

**Fig. 2:**
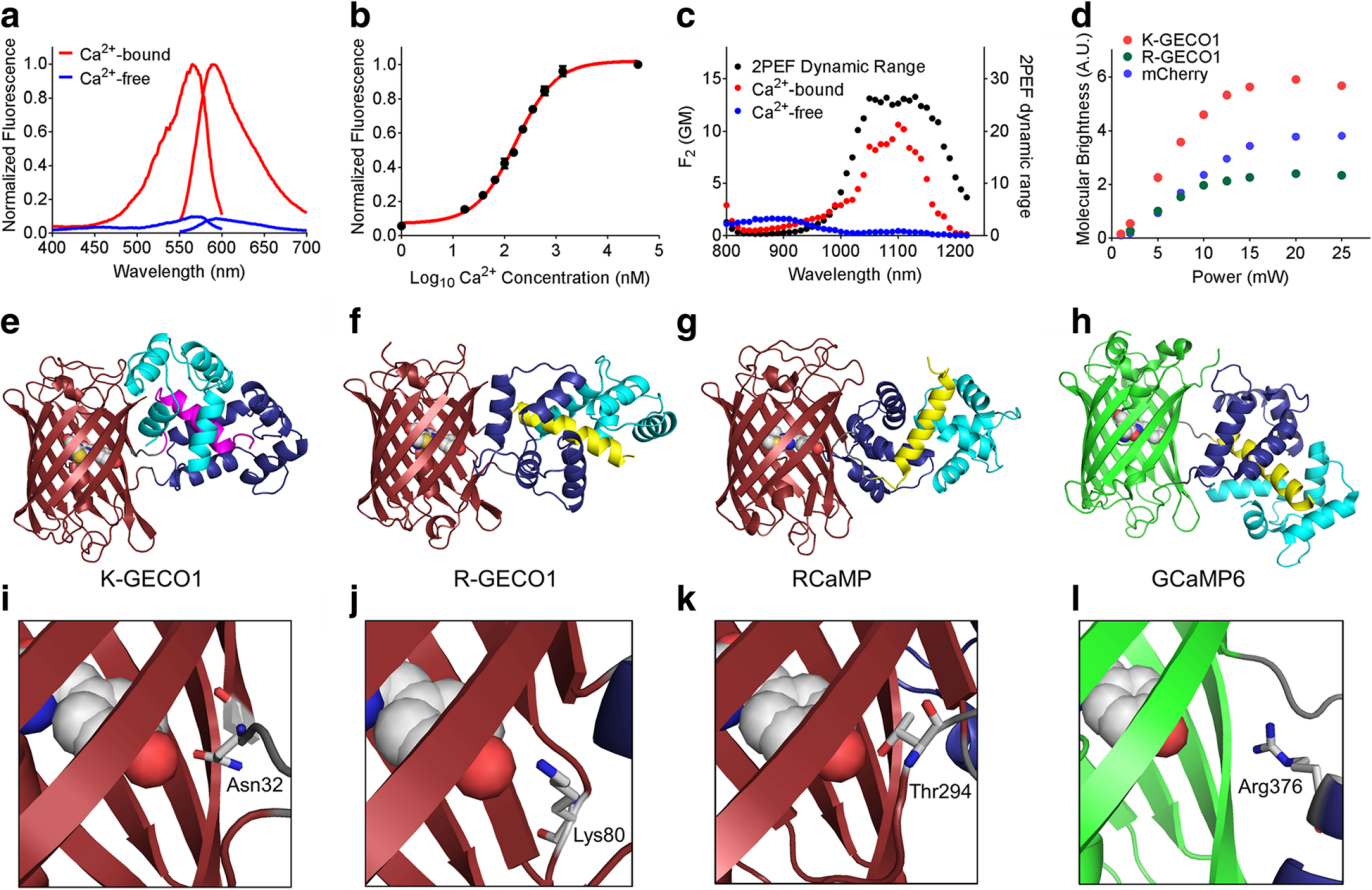
Characterization and structure of K-GECO1. **a** Fluorescence excitation and emission profile of K-GECO1 in the presence and absence of Ca^2+^. **b** Ca^2+^ titration curve of K-GECO1. **c** K-GECO1 effective two-photon fluorescence excitation spectra in Ca^2+^-saturated (red symbols) and Ca^2+^-free (blue symbols) states. Ratio of the K-GECO1 two-photon excitation fluorescence Ca^2+^-saturated/Ca^2+^-free signals as a function of wavelength (black symbols, plotted on right *y*-axis). **d** Two-photon molecular brightness of K-GECO1, R-GECO1, and mCherry with excitation at 1060 nm using various laser powers. Overall protein structures of genetically encoded Ca^2+^ indicators: **e** K-GECO1 (PDB: 5UKG), **f** R-GECO1 (PDB: 4I2Y [19]), **g** RCaMP (PDB: 3U0K [19]), and **h** GCaMP6 (PDB: 3WLD [60]), with ckkap colored in magenta, RS20 in yellow, the CaM N-lobe in dark blue, and the CaM C-lobe in cyan. Zoom-in view of the interactions between key residues and the chromophore: **i** K-GECO1, **j** R-GECO1, **k** RCaMP, and **l** GCaMP6. Supporting numeric data are provided in Additional file 8. PDB Protein Data Bank

The excitation and emission maxima of K-GECO1 are 568 and 594 nm, respectively, in the Ca^2+^-unbound state. In the Ca^2+^-bound state, these two maxima are slightly blue-shifted to 565 and 590 nm (Fig. 2a, Additional file 2: Table S1). K-GECO1 exhibits a 12-fold fluorescent intensity increase upon Ca^2+^ binding, with the extinction coefficient increasing from 19,000 to 61,000 M^-1^cm^-1^ and the quantum yield from 0.12 to 0.45 (Additional file 2: Table S1). The fluorescence spectra characteristics and Ca^2+^-induced fluorescence change of K-GECO1 are generally very similar to those of R-GECO1 (Additional file 2: Table S1). However, K-GECO1 is about twofold brighter than R-GECO1 under one-photon excitation. Ca^2+^ titration of purified K-GECO1 reveals that the protein has an apparent *K*_d_ of 165 nM with a Hill coefficient of 1.12 (Fig. 1b, Additional file 2: Table S1), similar to R-CaMP2 and other ckkap-based GECIs [23, 35].

K-GECO1 displayed moderate photoactivation when illuminated with either a 405-nm or 488-nm laser, in both the Ca^2+^-free and Ca^2+^-bound states. For Ca^2+^-bound K-GECO1, illuminating with 405-nm (1.76 W/cm^2^) or 488-nm (6.13 W/cm^2^) laser light for 1 s resulted in a ~20% increase in fluorescence as detected using 561-nm illumination. For Ca^2+^-free K-GECO1, 1 s of 405-nm (1.76 W/cm^2^) or 488-nm (6.13 W/cm^2^) laser light also resulted in a ~20% increase in fluorescence (Additional file 3: Figure S2a). Consistent with previous reports [19, 21], we observed a more pronounced photoactivation with R-GECO1, but not RCaMP1h, under similar illumination conditions (Additional file 3: Figure S2b–d).

K-GECO1 shows a strong two-photon excitation peak at approximately 1100 nm (Fig. 2c) in the Ca^2+^-bound state. A ~25-fold maximal increase of fluorescence signal, using two-photon excitation in the excitation region from 1050 to 1150 nm, occurs upon binding Ca^2+^ (Fig. 2c). The peak two-photon molecular brightness of K-GECO1 was compared with R-GECO1, using mCherry as a standard with 1060-nm excitation. The peak two-photon molecular brightness, defined as the maximum detected fluorescence count rate per emitting molecule [36], was obtained from the average fluorescence count rate and the average number of emitting molecules in the beam as determined by fluorescence correlation spectroscopy. Using this approach, K-GECO1 was found to be approximately 1.5-fold brighter than mCherry and over twofold brighter than R-GECO1 (Fig. 2d), which is consistent with the comparison of one-photon brightness for the Ca^2+^-bound state (Additional file 2: Table S1).

### Crystal structure of K-GECO1

To gain insight into the molecular mechanism of K-GECO1 Ca^2+^ sensitivity and to assist future protein engineering efforts, we determined the X-ray crystal structure of K-GECO1 in the Ca^2+^-bound form. The structure was determined to 2.36-Å resolution by molecular replacement (Fig. 2e, Additional file 4: Table S2). The crystal structure reveals the distinctive features of the ckkap/CaM complex in K-GECO1 (and presumably in other ckkap-based GECIs) relative to other RS20/CaM-based GECIs, including R-GECO1 (Fig. 2f), RCaMP (Fig. 2g), and GCaMP6 (Fig. 2h). The major difference is that the binding orientation of the ckkap peptide to the CaM domain is opposite to that of RS20 to CaM [37, 38]. Another difference is that the RS20 peptide consists entirely of an α-helix in the CaM-binding region, whereas the CaM-binding region of ckkap consists of both an α-helical segment as well as a hairpin-like loop structure at its C-terminus [35].

Examination of the molecular interactions between the protein and the chromophore at the circular permutation site provides insights into the mechanism of Ca^2+^-dependent fluorescence modulation. The side chain of Asn32 of linker1 is in direct hydrogen bonding with the phenolate oxygen of the chromophore (Fig. 2i), and is positioned similarly to Ser143 of FusionRed, which engages in a similar interaction with the chromophore [9]. We reason that Asn32 plays a critical role in communicating the Ca^2+^-dependent conformational change in the ckkap/CaM domain to the chromophore in the cpRFP domain. Lys79 of R-GECO1 (Fig. 2j), Thr243 of RCaMP1h (Fig. 2k), and Arg376 of GCaMP6 (Fig. 2l) are likely to have similar roles in their respective mechanisms of fluorescence modulation. Saturation mutagenesis of Asn32 of K-GECO1 resulted in a library of variants that all had dimmer fluorescence and/or a smaller Ca^2+^-induced fluorescence intensity fold change. These results indicate that Asn is the optimal residue in this position.

### Performance of K-GECO1 in cultured cells

**Fig. 3:**
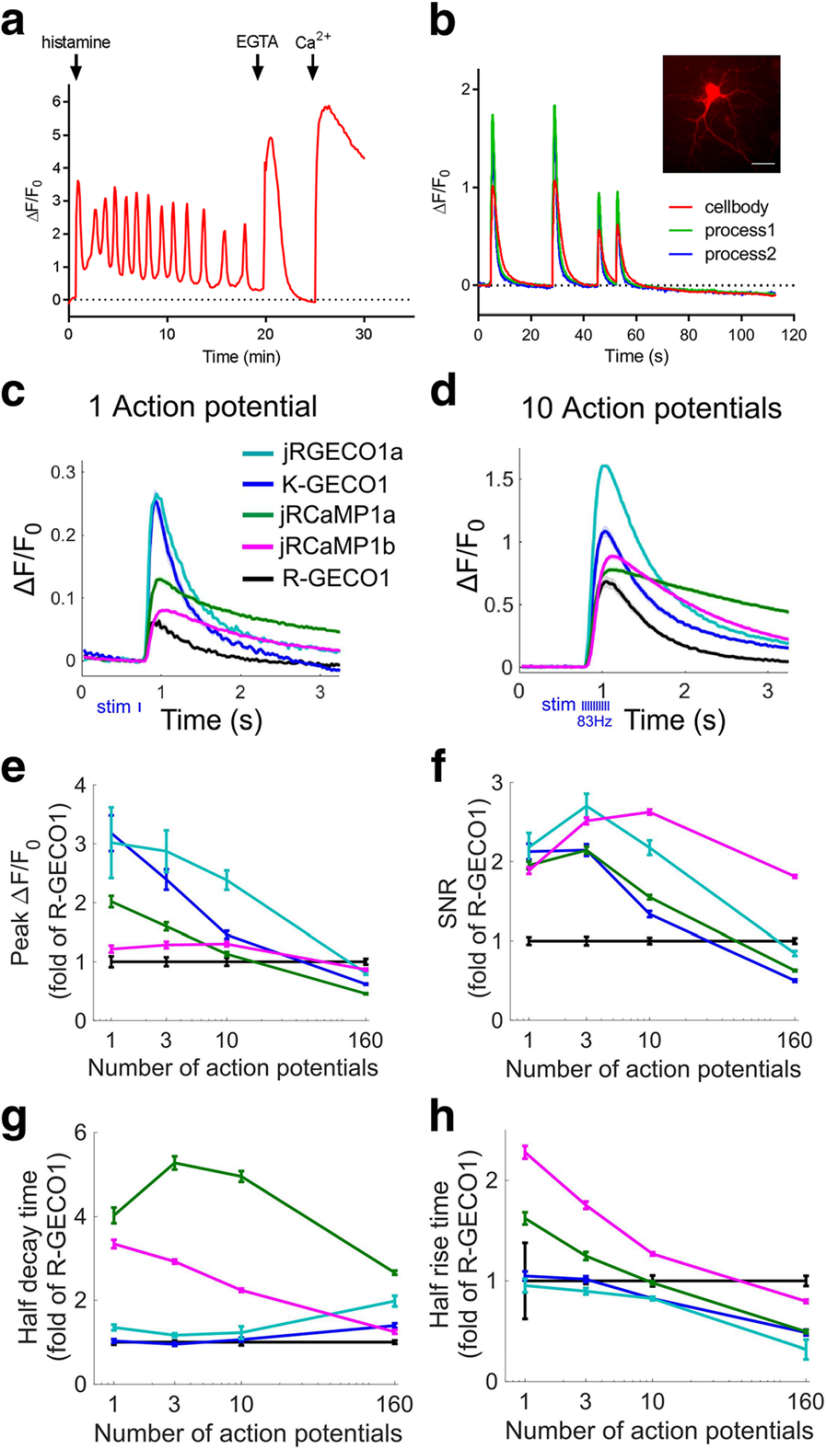
Performance of K-GECO1 in HeLa cells and cultured dissociated neurons. **a** Representative fluorescence time-course traces for HeLa cells expressing K-GECO1 with pharmacologically induced Ca^2+^ changes. **b** Imaging of spontaneous Ca^2+^ oscillations in dissociated neurons expressing K-GECO1. Inset: Fluorescence image of dissociated neurons expressing K-GECO1 (scale bar, 30 μm). **c** Average responses for one action potential for K-GECO1 compared with other red GECIs (the same color code is used in panels **c**–**h**). **d** Responses of ten action potentials of red GECIs. **e**–**h** Comparison of K-GECO1 and other red GECIs as a function of number of action potentials. **e** Response amplitude, Δ*F*/*F*_0_. **f** Signal-to-noise ratio (SNR). **g** Half decay time. **h** Half rise time. For (**e**–**h**), *n* = 56 wells, 827 neurons for K-GECO1; *n* = 66 wells, 1029 neurons for R-GECO1; *n* = 38 wells, 682 neurons for jRGECO1a; *n* = 105 wells, 2420 neurons for jRCaMP1a; *n* = 94 wells, 2995 neurons for jRCaMP1b. Supporting numeric data are provided in Additional file 9. GECI genetically encoded Ca^2+^ indicator, SNR signal-to-noise ratio

To demonstrate the utility of K-GECO1 in imaging Ca^2+^ dynamics, we expressed it in cultured human cells, dissociated rat neurons, organotypic rat brain slices, zebrafish sensory neurons, and mouse primary visual cortex. We first recorded the response of K-GECO1 to changes in the cytoplasmic Ca^2+^ concentration in HeLa cells using established protocols (Fig. 3a) [39]. HeLa cells expressing K-GECO1 had maximum fluorescence intensity changes of 5.2 ± 1.1-fold (*n* = 44) on treatment with histamine, which is similar to the 4.9 ± 1.9-fold (*n* = 22) response previously reported for R-GECO1 expressing HeLa cells [18].

Next, we tested K-GECO1 in dissociated rat hippocampal neurons. The relatively low Ca^2+^ *K*_d_ of 165 nM for K-GECO1 is comparable to that of current best green GECI, GCaMP6s [17], which has been highly optimized for detection of neuronal Ca^2+^ transients. Cultured dissociated neurons expressing K-GECO1 had fluorescence distributed throughout the cytosol and nucleus, and exhibited close to twofold maximum increases for spontaneous Ca^2+^ changes (Fig. 3b). We did not observe intracellular fluorescent punctate structures, as have been observed for R-GECO1 and its variants [22, 27], in the cell bodies of dissociated neurons expressing K-GECO1 (Additional file 5: Figure S3a,b). We also did not observe noticeable photoactivation of K-GECO1 in neurons when illuminated with 0.5 W/cm^2^ of 405-nm laser light. Under the same illumination conditions, R-GECO1 exhibited substantial photoactivation (Additional file 5: Figure S3c,d). The absence of photoactivation for K-GECO1 under these conditions might be due to the relative low laser intensity (0.5 W/cm^2^) compared with the intensity (1.76 W/cm^2^) used for in vitro characterization.

To compare the performance of K-GECO1 with other red GECIs in dissociated neurons, we performed an automated imaging assay with field stimulation as previously described [17, 24]. For a single action potential, K-GECO1 exhibited a similar response to jRGECO1a (Fig. 3c) and GCaMP6s [17], two of the most sensitive indicators currently available. The peak Δ*F*/*F*_0_ amplitude of K-GECO1 with three or more action potentials was smaller than that of jRGECO1a, yet better than other red GECIs (Fig. 3d,e). In terms of the signal-to-noise ratio, K-GECO1 had similar performance to jRGECO1a, but less than that of jRCaMPa/b (Fig. 3f). K-GECO1 exhibits fast kinetics, with a half decay time that is faster than jRGECO1a and jRCaMP1a/b (Fig. 3g), and a half rise time that is similar to jRGECO1a but faster than jRCaMP1a/b (Fig. 3h).

**Fig. 4:**
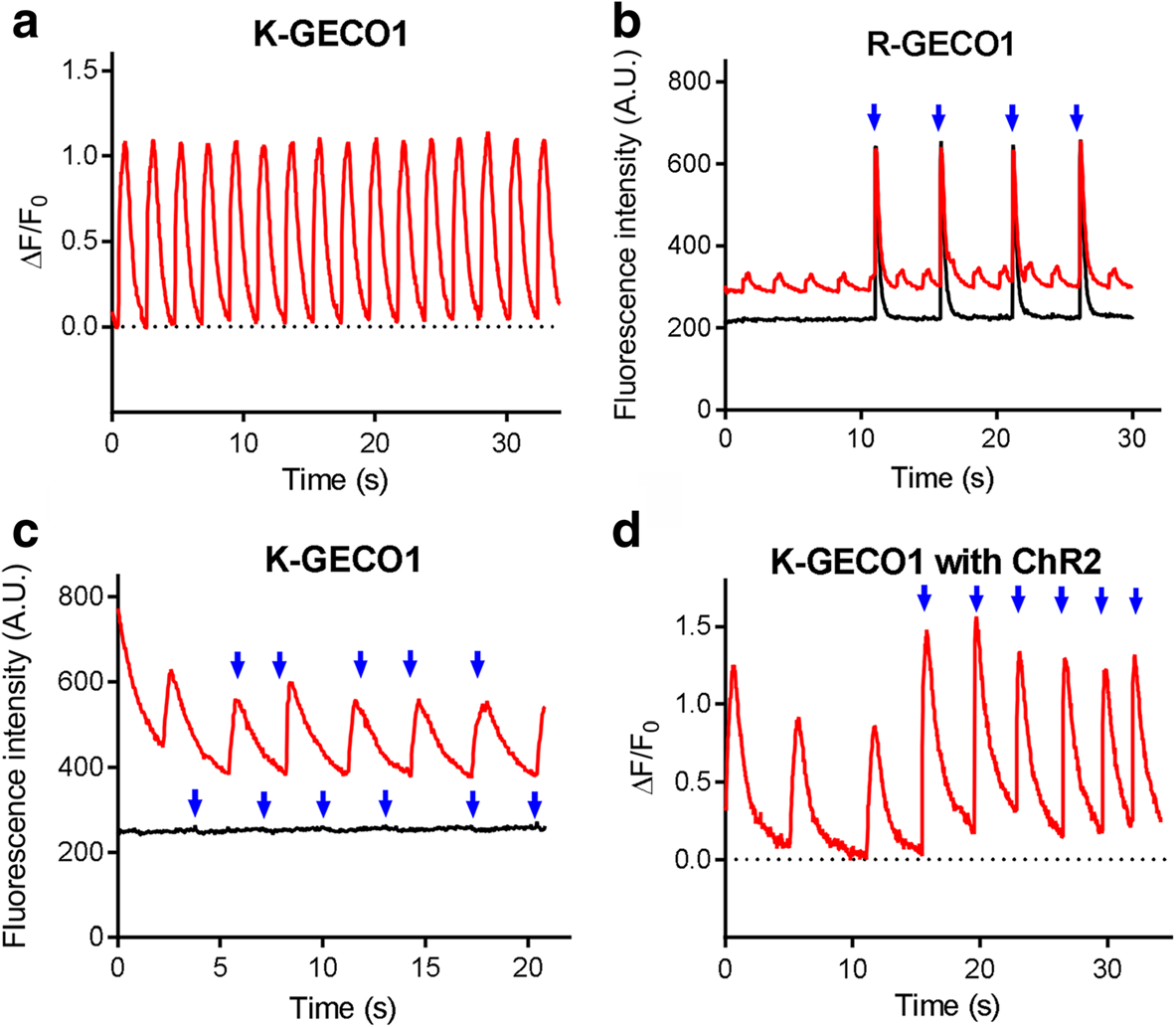
Performance of K-GECO1 in iPSC-CMs. **a** Representative time course of spontaneous Ca2+ oscillations in iPSC-CMs as imaged using K-GECO1. **b** Photoactivation of R-GECO1 and **c** K-GECO1 in iPSC-CMs. Cells with spontaneous activity are colored in red and cells with no spontaneous activity are colored in black. **d** Combined use of K-GECO1 with ChR2. Illumination with 150 ms of 470-nm light is indicated by blue arrowheads. Supporting numeric data are provided in Additional file 10. A.U. arbitrary units, ChR2 channel rhodopsin-2, iPSC-CM induced pluripotent stem cell–derived cardiomyocyte

As our in vitro characterization indicated that K-GECO1 has less blue-light photoactivation than R-GECO1, we tested its performance in human induced pluripotent stem cell–derived cardiomyocytes (iPSC-CMs) in combination with channel rhodopsin-2 (ChR2). As expected, transfected iPSC-CMs expressing K-GECO1 exhibited spontaneous Ca^2+^ oscillations (Fig. 4a). To compare photoactivation of K-GECO1 and R-GECO1 in iPSC-CMs, we illuminated transfected cells (GECI only, no ChR2) with 0.19 W/cm^2^ of 470-nm LED light (Fig. 4b,c). Under these conditions, R-GECO1 exhibited a substantial photoactivation effect with a transient 200% increase in red fluorescence. Under the same illumination conditions, K-GECO1 had a negligible change in red fluorescence. When we co-transfected iPSC-CMs with both K-GECO1 and ChR2, blue-light stimulation reliably induced Ca^2+^ transients (Fig. 4d), demonstrating that the combination of K-GECO1 and ChR2 is feasible for all-optical excitation and imaging of iPSC-CMs.

### Performance of K-GECO1 in organotypic brain slices

**Fig. 5:**
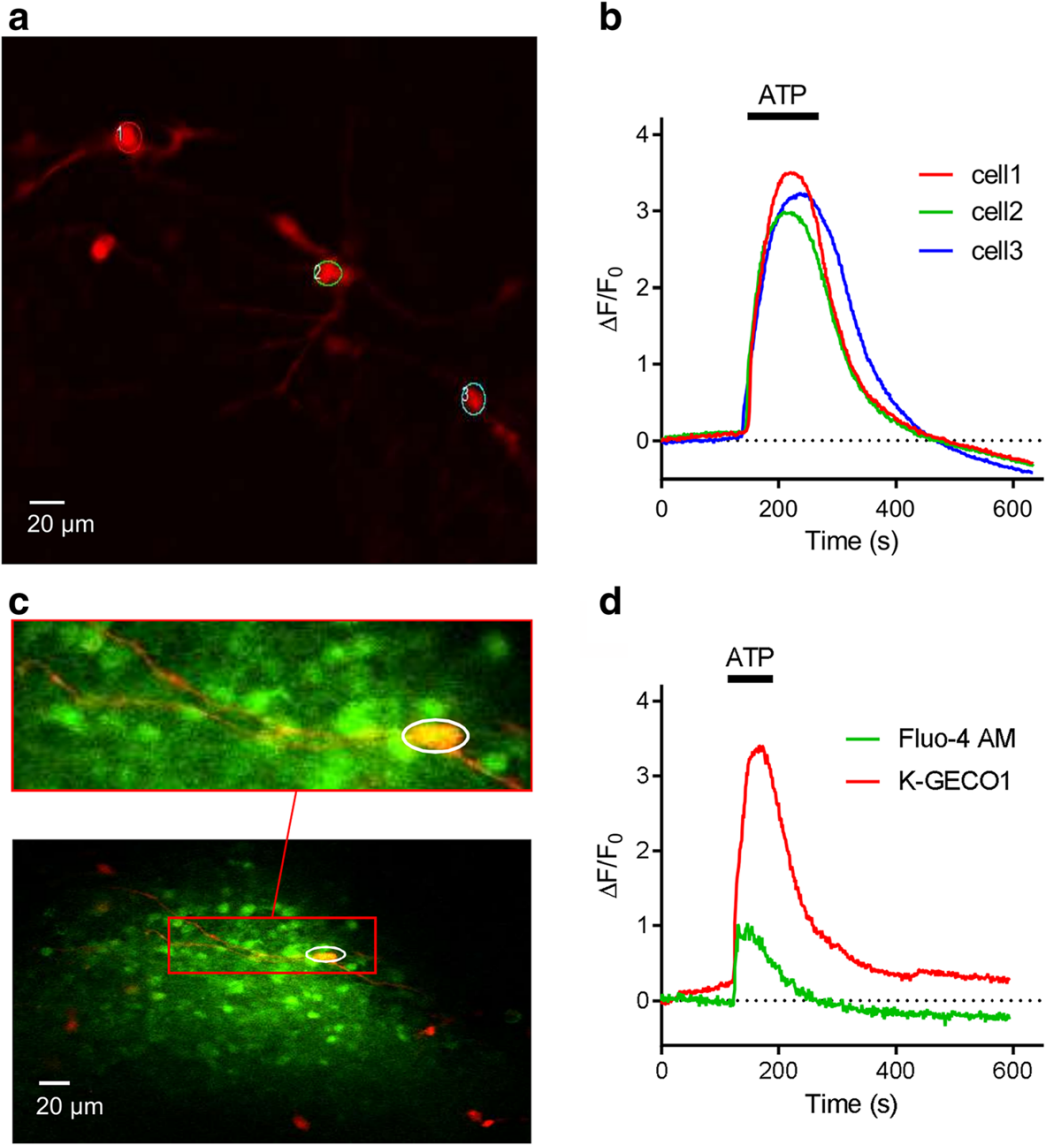
Performance of K-GECO1 in organotypic brain slices. **a** K-GECO1 labeling of the soma and dendrites of neurons in the ventromedial nucleus (VMN) of organotypically cultured newborn rat hypothalamus slices. **b** ATP-induced cytosolic Ca^2+^ rise in VMN neurons. **c** Fluo-4 AM loaded and K-GECO1 transfected into VMN slice. **d** Representative fluorescence intensity traces of ATP treatment causing a Ca^2+^ rise, as reported by both Fluo-4 AM and K-GECO1. Supporting numeric data are provided in Additional file 11. ATP adenosine triphosphate, VMN ventromedial nucleus

We further tested the performance of K-GECO1 by expressing it in organotypic slices of the newborn rat ventromedial nucleus (VMN) of the hypothalamus. Expression of K-GECO1 enabled visualization of both neuronal cell bodies and processes (Fig. 5a). We investigated the performance of K-GECO1 under pharmacological stimulation by adenosine triphosphate (ATP) (100 μM), which activates suramin-sensitive ATP receptors and induces an influx of extracellular Ca^2+^, thus increasing the cytosolic Ca^2+^ concentration [40]. Upon treatment with ATP, neurons expressing K-GECO1 underwent a mean increase in fluorescence intensity of 3.26 + 0.18-fold (*n* = 21) (Fig. 5b).

To compare the performance of K-GECO1 with the small molecule-based green cytosolic Ca^2+^ indicator, Fluo-4 AM, we loaded the dye into VMN neurons that were expressing K-GECO1 (Fig. 5c). When treated with ATP, these neurons (*n* = 3) exhibited a 3.01 + 0.86-fold increase in K-GECO1 fluorescence, but only a 0.70 + 0.12-fold increase in Fluo-4 fluorescence (Fig. 5d). In non-transfected cells stained with Fluo-4 AM, we did not observe any crosstalk from Fluo-4 AM into the red channel. Overall, K-GECO1 unravels robust responses to cytosolic Ca^2+^ concentration changes in neurons in organotypic brain slices.

### In vivo Ca^2+^ imaging with K-GECO1

**Fig. 6:**
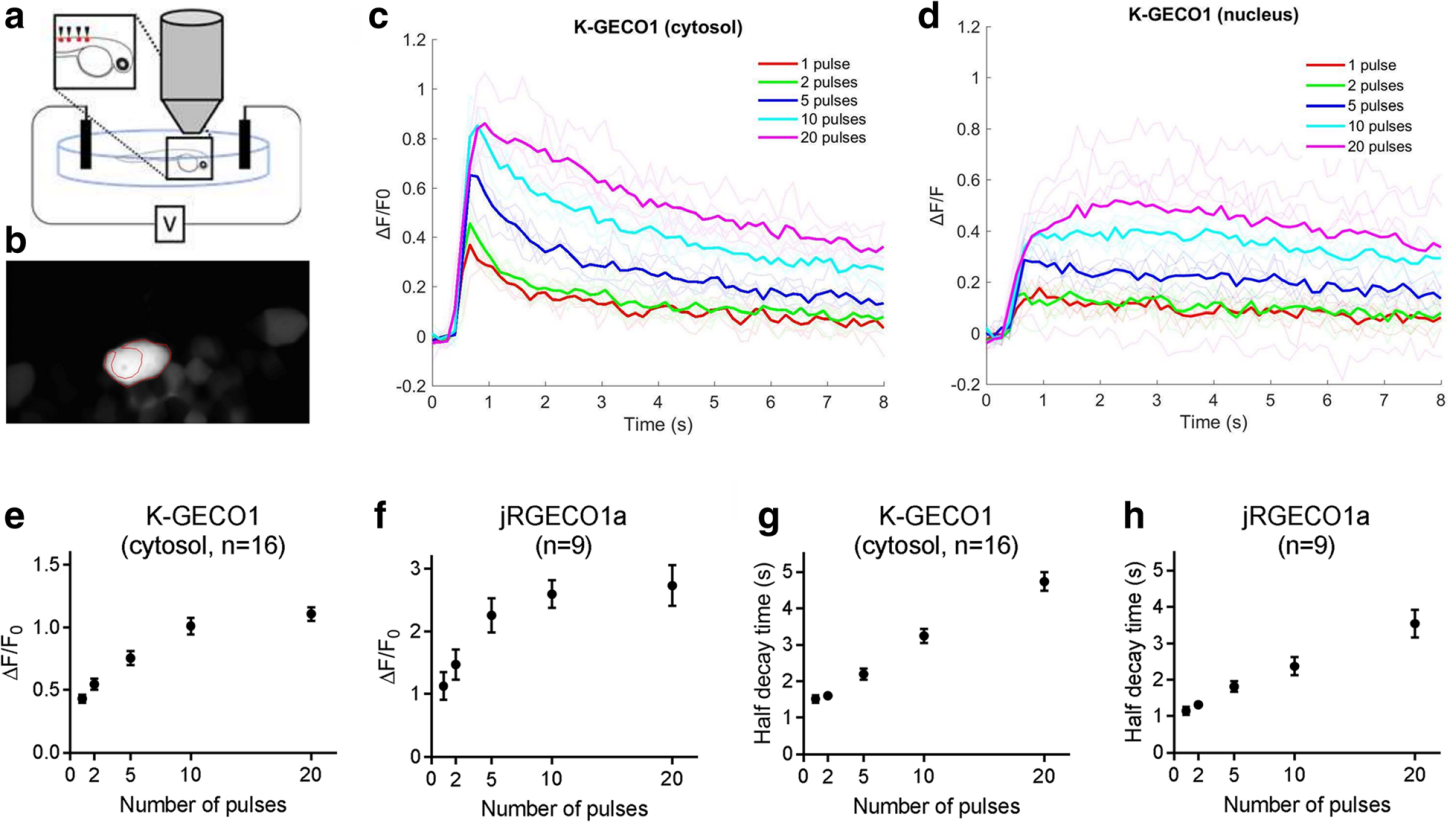
In vivo imaging of K-GECO in zebrafish Rohon–Beard cells. **a** Schematic setup of the experiment. **b** Image of Rohon–Beard cells expressing K-GECO1 with region of interest (ROI) indicating cytoplasm. **c** K-GECO1 Ca^2+^ response to pulse stimuli in the cytosol. **d** K-GECO1 Ca^2+^ response to pulse stimuli in the nucleus. **e** Fluorescence fold change of K-GECO1 and **f** jRGECO1a under various numbers of pulses. **g** Half decay time of K-GECO1 and **h** jRGECO1a under various numbers of pulses. Supporting numeric data are provided in Additional file 12

To test K-GECO1 in zebrafish spinal cord sensory neurons in vivo, we transiently expressed K-GECO1 in Rohon–Beard (RB) cells. Zebrafish RB cells have previously been used for in vivo GECI imaging and shown to fire a single spike in response to each electrical pulse to the skin [41]. Electrical stimulations were applied to trigger Ca^2+^ transients at 3 days post fertilization. Two-photon imaging with excitation at 1140 nm (Fig. 6a) revealed that K-GECO1 filled both the cytoplasm and nucleus in vivo in zebrafish RB neurons (Fig. 6b). Cytoplasmic K-GECO1 exhibited a ~40% fluorescence intensity increase to Ca^2+^ transients triggered by a single pulse stimulus (Fig. 6c). When the RB neurons were stimulated with 5 to 20 repetitive stimuli, 50–100% increases in K-GECO1 fluorescence were observed (Fig. 6d). As expected, the fluorescence response in the nucleus was diminished with respect to the response in the cytosol, and exhibited a slower recovery to baseline (Fig. 6c,d). Compared to the optimized red fluorescent indicator jRGECO1a, K-GECO1 showed decreased sensitivity in zebrafish in terms of stimulus-evoked fluorescence change (Fig. 6e,f), whereas the half decay time was comparable (Fig. 6g, h). Consistent with the results from dissociated neurons, an even distribution of the K-GECO1 red fluorescence in RB cells was observed in zebrafish neurons in vivo (Additional file 6: Figure S4a,b), while jRGECO1 exhibited fluorescence accumulations (Additional file 6: Figure S4c).

**Fig. 7:**
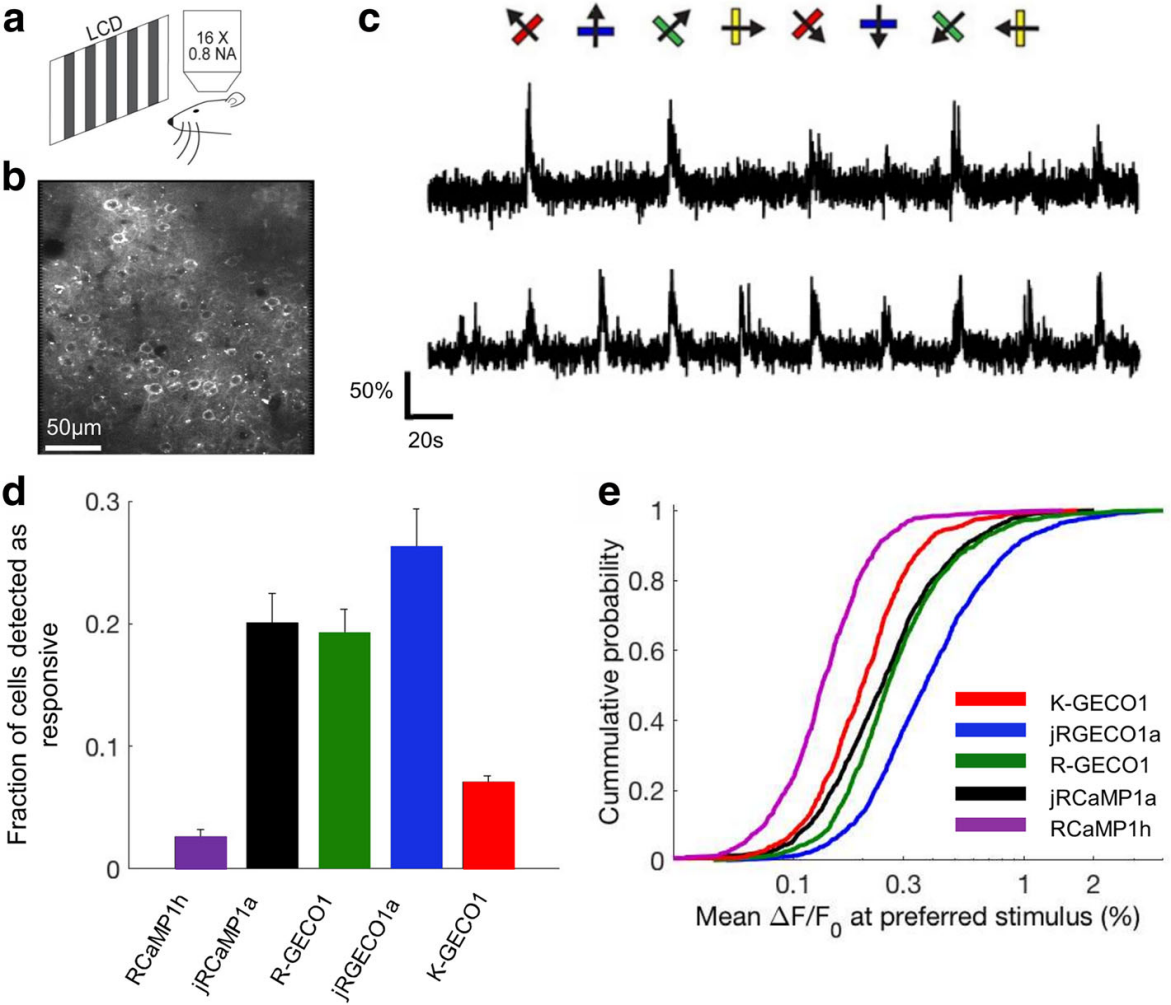
In vivo imaging of K-GECO1 in mouse V1 neurons. **a** Schematic setup of the experiment. **b** Image of V1 L2/3 cells expressing K-GECO1. **c** Example traces from neurons expressing K-GECO1. The direction of grating motion is indicated above the traces. **d** Fraction of cells detected as responding to the visual stimulus of K-GECO1 compared with previously reported values [24] from other red GECIs (*n* = 26 for RCaMP1h; *n* = 45 for jRCaMP1a; *n* = 30 for R-GECO1; *n* = 40 for jRGECO1a; *n* = 13 for K-GECO1). **e** Distribution of Δ*F*/*F*_0_ amplitude for the preferred stimulus of K-GECO1 compared with previously reported values [24] from other red GECIs. GECI genetically encoded Ca^2+^ indicator

To evaluate K-GECO1 in the mouse primary visual cortex (V1) in vivo, V1 neurons were infected with adeno-associated virus (AAV) expressing nuclear export signal (NES) tagged K-GECO1 under the human synapsin-1 promoter (AAV-SYN1-NES-K-GECO1). The majority of V1 neurons can be driven to fire action potentials in response to drifting gratings. Eight-direction moving grating visual stimuli were presented to the contralateral eye (Fig. 7a). K-GECO1 expressing L2/3 neurons exhibited cytoplasmic red fluorescence (Fig. 7b), and two-photon imaging revealed visual-stimulus-evoked fluorescence transients in subsets of neurons (Fig. 7c). We compared the performance of K-GECO1 with other red GECIs using previously established metrics [17, 24]. The fraction of neurons detected as responsive in the visual cortex is higher for K-GECO1 than RCaMP1h, but lower than R-GECO1 and other optimized red indicators (Fig. 7d). The mean Δ*F*/*F*_0_ at the preferred visual stimulus is reflective of indicator sensitivity. By this metric, K-GECO1 has sensitivity that is comparable to those of R-GECO1 and jRCaMP1a, but less than jRGECO1a (Fig. 7e). Lysosomal accumulation was previously observed in mouse V1 neurons labeled with jRGECO1a, but not in the ones with jRCaMP1a/b [24]. Fixed brain tissue sections, prepared as previously reported for jRGECO1a and jRCaMP1a/b [24], revealed no signs of lysosomal accumulation in K-GECO1-expressing V1 neurons (Additional file 7: Figure S5a). As with both jRGECO1a and jRCaMP1a/b, in vivo functional imaging of K-GECO1 did exhibit fluorescent clump-like structures (Additional file 7: Figure S5b), yet these structures were not observed in fixed sections of the same tissue. We are currently unable to explain this discrepancy. Overall, the results demonstrate that K-GECO1 can be used to report physiological Ca^2+^ changes in neurons in vivo with performance that matches or surpasses that of other first-generation red fluorescent Ca^2+^ indicators.

## Discussion

Although green fluorescent GECIs are currently the most highly effective tools for in vivo visualization of neuronal signaling, we anticipate that they will one day be made redundant by red fluorescent GECIs due to the inherent advantages associated with longer wavelength fluorescence. The transmittance of tissue increases as the wavelength increases, so red fluorescent GECIs will enable imaging of neuronal activity deeper into brain tissue than is possible with green fluorescent GECIs, assuming all other properties are equivalent [24, 30]. In addition, red fluorescent GECIs enable multiparameter imaging in conjunction with green fluorescent indicators, and facilitate simultaneous imaging and optical activation when used in conjunction with blue-light activatable optogenetic actuators such as ChR2 [42]. However, as widely recognized [13, 19, 22, 24], red GECIs currently suffer from a number of limitations compared to the most highly optimized green GECIs (i.e., GCaMP6) [17]. These limitations include decreased sensitivity for RCaMP variants and complicated photophysics and lysosomal accumulation for R-GECO variants. As both green and red GECIs have analogous designs and contain identical Ca^2+^-binding domains, these undesirable characteristics are related to the RFP scaffold used to generate red GECIs.

To overcome the limitations associated with current RFP scaffolds, we turned our attention to the eqFP578-derived lineage of monomeric RFPs (i.e., mKate and its derivatives) [7–9], which tend to give bright and evenly distributed fluorescence when expressed in the neurons of transgenic mice [31]. Using a semi-rational design and directed evolution, we developed a new red fluorescent Ca^2+^ indicator, K-GECO1, based on the mKate variant FusionRed [9]. We anticipated that K-GECO1 would retain the favorable traits associated with its starting template RFP. We found this expectation to be generally true, as we did not observe lysosomal aggregation in dissociated rat neurons, zebrafish neurons, or fixed mouse brain tissue expressing K-GECO1. Some fluorescent punctate-like structures were observed during in vivo functional imaging.

The other distinctive feature of K-GECO1 is the use of the ckkap peptide as the CaM binding partner for the Ca^2+^-binding motif. Consistent with previous reports [23, 35], the ckkap/CaM motif yielded a lower apparent *K*_d_ for Ca^2+^ and faster kinetics (relative to RS20/CaM), and an apparent Hill coefficient close to 1. These characteristics should enable more sensitive detection of Ca^2+^ dynamics at physiological ranges, as is evident from K-GECO1’s large fluorescence response amplitude for a single action potential. With a Hill coefficient close to 1, K-GECO1 should provide a more linear Ca^2+^ response following multiple stimuli.

The X-ray crystal structure of K-GECO1 suggests that the indicator has a self-contained fluorescence modulation mechanism, similar to that proposed for R-GECO1 [22, 29]. Unlike GCaMP, in which the fluorescence modulation mechanism is dependent on the interactions with a residue of CaM [43] (Fig. 2l), the K-GECO1 Ca^2+^-bound state is likely stabilized by the hydrogen bonding between the phenolate group of chromophore and linker1 residue Asn32 (Fig. 2i). This makes the cpFusionRed protein in K-GECO1 a potentially useful template as a signal transduction domain to be combined with other binding domains for development of new types of red fluorescent indicators. The crystal structure also reveals that the ckkap/CaM motif in K-GECO1 has a reversed binding orientation for CaM compared with the RS20/CaM binding patterns in R-GECO1, RCaMP, and GCaMP6 (Fig. 2e–h). These results indicate that the GCaMP-like design is versatile enough to tolerate different peptide conformations and CaM orientations, and that exploring a wider range of CaM binding partners is likely to lead to GECIs with new and improved properties.

First-generation red GECIs, including mApple-based R-GECO1 and mRuby-based RCaMP1h, have been optimized using a neuron screening platform [24, 44], resulting in jRGECO1a and jRCaMP1a/b with greatly improved in vivo performance for detection of action potentials. Although K-GECO1 is a first-generation red GECI, it already provides performance that, by some criteria, is comparable to second-generation red GECIs. Specifically, K-GECO1 has a fluorescent response to single action potentials that is similar to that of jRGECO1a (and superior to jRCaMP1a/b) and faster dissociation kinetics than either jRGECO1a or jRCaMP1a/b. However, by other criteria, K-GECO1 will require further optimization to match the performance of second-generation red GECIs. For example, K-GECO1 does not provide the same level of in vivo sensitivity as the highly optimized jRGECO1a. In addition, K-GECO1 showed some blue-light-dependent photoactivation during in vitro characterization, though less so than R-GECO1. The photoactivation of K-GECO1 was not detectable under the illumination condition in our characterizations in cultured dissociated neurons (Additional file 5: Figure S3c) or in iPSC-CMs (Fig. 4c), suggesting that it is more suitable than R-GECO1 for use with blue/cyan-excitable optogenetic actuators. Nevertheless, the occurrence (or absence) of photoactivation will depend on the specific illumination conditions, and so appropriate controls (i.e., blue-light illumination of tissue expressing K-GECO1 but no optogenetic actuator) must be performed. Future efforts to screen K-GECO variants in neuronal cells, as was done for R-GECO1 and RCaMP1h [24], could lead to the discovery of improved variants with higher levels of expression in neurons, *K*_d_s tuned to the range of neuronal cytoplasmic Ca^2+^ concentrations, increased cooperativity of Ca^2+^ binding to improve single action potential detection, diminished lysosomal accumulation, and minimum blue-light activation.

## Conclusion

In summary, we have demonstrated the utility of K-GECO1 in various cell types including HeLa cells, dissociated neurons, iPSC-CMs, neurons in organotypic rat brain slices, zebrafish RB cells, and mouse V1 neurons in vivo. Though not yet ideal by all criteria, K-GECO1 represents a step forward in the development of red GECIs. Current users of red GECIs may find switching to K-GECO1 advantageous if their applications would benefit from faster kinetics, a more linear fluorescent response to multiple stimuli, or decreased photoactivation with blue-light illumination. For new users, we suggest performing initial trials with several different indicators to decide which one offers the best performance for their application. Due to the differences in expression and accumulation associated with red fluorescent proteins from different species and in different cellular contexts, new users should try one DsRed-derived GECI (e.g., jRGECO1a or R-CaMP2) [23, 24], one eqFP611-derived GECI (e.g., jRCaMP1a/b) [24], and one eqFP578-derived GECI (e.g., K-GECO1). As with R-GECO1 and RCaMP1h, further optimization using a neuron-based screening approach is likely to yield K-GECO variants with much improved sensitivity and performance in vivo.

## Methods

### Protein engineering

The design of K-GECO is based on well-established GECI designs reported previously [18, 33, 45–47]. The initial construction of mKate2 and FusionRed-based Ca^2+^ indicators was done by overlapping the assembly of the four DNA parts encoding the following protein fragments: the N-terminal (1–145) and C-terminal (146–223) parts of mKate2 or FusionRed, the RS20 peptide, and the CaM of R-GECO1. The fragments were amplified by PCR from mKate2, FusionRed (a kind gift from Michael Davidson), and R-GECO1 DNA. The overlap region and restriction sites were encoded in the primers. DNA encoding ckkap was synthesized by Integrated DNA Technologies (IDT). Purified PCR products were pooled and assembled in an overlapping PCR reaction. The resulting assembled PCR product was purified, digested with *Xho*I and *Hind*III (Thermo Fisher Scientific), and then ligated into a similarly digested pBAD/His B vector (Thermo Fisher Scientific). The ligation product was transformed into electrocompetent *E. coli* strain DH10B cells. Plasmids were purified with the GeneJET miniprep kit (Thermo Fisher Scientific) and then sequenced using the BigDye Terminator Cycle Sequencing kit (Thermo Fisher Scientific).

EP-PCR amplifications were performed to construct random mutagenesis libraries. The EP-PCR products were digested with *Xho*I and *Hind*III, and then ligated into a similarly digested pBAD/His B vector (Thermo Fisher Scientific). To construct site-directed mutagenesis and saturation mutagenesis libraries, QuikChange site-directed mutagenesis Lightning Single or Multi kit (Agilent Technologies) was used according to the manufacturer's instructions. The resulting variant libraries were transformed into electrocompetent *E. coli* strain DH10B cells and incubated overnight at 37 °C on 10-cm petri dishes with lysogeny broth (LB) agar supplemented with 400 g/mL ampicillin (Sigma) and 0.02% (wt/vol) L-arabinose (Alfa Aesar).

A custom imaging system was used for screening K-GECOs on plate with *E. coli* colonies expressing the variants [48]. When screening, fluorescence images of *E. coli* colonies were taken for each petri dish with an excitation filter of 542/27 nm and an emission filter of 609/57 nm. The colonies with the highest fluorescence intensity in each image were then picked and cultured in 4 mL liquid LB medium with 100 μg/ml ampicillin and 0.02% L-arabinose at 37 °C overnight. Proteins were then extracted using B-PER reagents (Thermo Fisher Scientific) from the liquid culture. The protein extraction was used for a secondary screen of the Ca^2+^-induced response test using Ca^2+^-free buffer (30 mM 3-(N-morpholino)propanesulfonic acid (MOPS), 100 mM KCl, and 10 mM EGTA at pH 7.2) and Ca^2+^-buffer (30 mM MOPS, 100 mM KCl, and 10 mM Ca-EGTA at pH 7.2) in a Safire2 fluorescence microplate reader (Tecan).

### In vitro characterization

To purify K-GECO variants for in vitro characterization, the pBAD/His B plasmid encoding the variant of interest was used to transform electrocompetent *E. coli* DH10B cells and then plated on LB-agar plate with ampicillin (400 μg/mL). Single colonies were picked and inoculated into 5 mL LB medium supplemented with 100 g/mL ampicillin. Bacterial subcultures were incubated overnight at 37 °C. Then, 5 mL of bacterial subculture was added into 500 mL of LB medium with 100 μg/mL of ampicillin. The cultures were incubated at 37 °C to an OD of 0.6. Following induction with L-arabinose to a final concentration of 0.02% (wt/vol), the cultures were then incubated at 20 °C overnight. Bacteria were harvested by centrifugation at 4000 *g* for 10 min, resuspended in 30 mM Tris-HCl buffer (pH 7.4), lysed using a French press, and then clarified by centrifugation at 13,000 *g* for 30 mins. Proteins were purified from the cell-free extract by Ni-NTA affinity chromatography (MCLAB). The buffer of purified proteins was exchanged into 10 mM MOPS, 100 mM KCl, pH 7.2. Absorption spectra were recorded on a DU-800 UV-visible spectrophotometer (Beckman) and fluorescence spectra were recorded on a Safire2 fluorescence plate reader (Tecan).

To determine the quantum yield, the fluorescent protein mCherry was used as a standard. The detailed protocol has been described previously [18]. Briefly, the fluorescence emission spectra of each dilution of the protein solution of mCherry and K-GECO variants were recorded. The total fluorescence intensities were obtained by integration. The integrated fluorescence intensity versus absorbance was plotted for both mCherry and K-GECOs. The quantum yield was determined from the slopes of mCherry and K-GECOs. The extinction coefficient was determined by first measuring the absorption spectrum of K-GECO variants in a Ca^2+^-free buffer and a Ca^2+^-buffer. The absorption was measured following alkaline denaturation. The protein concentration was determined with the assumption that the denatured chromophore has an extinction coefficient of 44,000 M^-1^ cm^-1^ at 446 nm. The extinction coefficient of K-GECO variants was calculated by dividing the peak absorbance maximum by the concentration of protein.

For the Ca^2+^ *K*_d_ determination, the purified protein solution was diluted into a series of buffers, which were prepared by mixing Ca^2+^-buffer and Ca^2+^-free buffer with free Ca^2+^ concentration in a range from 0 to 3900 nM. The fluorescence intensity of K-GECO variants in each solution was measured and subsequently plotted as a function of Ca^2+^ concentration. The data were fitted to the Hill equation to obtain *K*_d_ and the apparent Hill coefficient.

Two-photon excitation spectra and cross sections were measured as previously reported [49], with the following adjustments. For the two-photon excited spectra (2PE), the fluorescence was collected through a 694/SP filter for K-GECO1 (Semrock). To correct for wavelength-to-wavelength variations in the laser parameters, a correction function using rhodamine B in MeOH and its known 2PE spectrum was applied [50]. Two-photon cross sections were measured at 1100 nm for K-GECO1, with rhodamine B in MeOH as a reference standard. The fluorescence for cross sections were collected through a narrow bandpass filter, 589/15 (Semrock), and differential quantum efficiencies were obtained at 582 nm with a PC1 ISS spectrofluorimeter (this wavelength corresponded to the bandpass center of the above filter when used in the MOM Sutter Instruments microscope due to its tilted position). Since the filter (694/SP) used for the 2PE spectra measurements covers the fluorescence of both the neutral and anionic forms of the chromophore, the spectrum of a particular Ca^2+^ state of a protein represents a combination of the unique 2PE spectra of the neutral and anionic forms, weighted to their relative concentrations (ρ, the concentration of one form divided by the total chromophore concentration) and quantum yields. The *y*-axis of the total 2PE spectrum is defined by *F*_2_(λ) = σ_2_,_N_(λ) φ_N_ ρ_N_ + σ_2_,_A_(λ) φ_A_ ρ_A_, where σ_2_(λ) is the wavelength-dependent two-photon cross section and φ is the fluorescence quantum yield of the corresponding form (N for neutral or A for anionic in the subscript). At the wavelengths used to measure the cross sections (1060 and 1100 nm), σ_2_,_N_ is assumed to be zero, and φ_A_ and ρ_A_ were independently measured to give a value for *F*_2_ (Goeppert-Mayer, GM). The relative concentrations of the neutral and anionic forms were found by measuring the absolute extinction coefficients of each respective form in the Ca^2+^-free and the Ca^2+^-bound states. These differ from the effective extinction coefficients reported in Additional file 2: Table S1, which are weighted by the relative concentrations of both forms of the chromophore.

For the fluorescence correlation spectroscopy measurement of two-photon molecular brightness, dilute protein solutions (50–200 nM) in Ca^2+^ buffer (30 mM MOPS, 100 mM KCl, 10 mM CaEGTA, pH 7.2) were excited at 1060 nm at laser powers from 1 to 25 mW for 200 s. At each laser power, the fluorescence was recorded by an avalanche photodiode and fed to an Flex03LQ autocorrelator (Correlator.com). The measured autocorrelation curve was fitted to a simple diffusion model with a custom Matlab program [36] to determine the average number of excited molecules 〈*N*〉 in the excitation volume. The two-photon molecular brightness (ε) at each laser power was calculated as the average rate of fluorescence 〈*F*〉 per emitting molecule 〈*N*〉, defined as *ε* = 〈*F*〉/〈*N*〉 in kilocounts per second per molecule. As a function of laser power, the molecular brightness initially increases as the square of the laser power, then levels off and decreases due to photobleaching or saturation of the protein chromophore in the excitation volume. The maximum or peak brightness achieved, 〈*e*_max_〉, represents a proxy for the photostability of a fluorophore.

To measure the photoswitching of K-GECO1, R-GECO1, and RCaMP1h in vitro, the purified protein in Ca^2+^ buffer (30 mM MOPS, 100 mM KCl, 10 mM CaEGTA, pH 7.2) or EGTA buffer (30 mM MOPS, 100 mM KCl, 10 mM EGTA, pH 7.2) were made into aqueous droplets with octanol in a 1:9 ratio and mounted on a presilanized coverslip. A single droplet was focused under the AxioImager microscope (Zeiss) with a 20× 0.8 NA objective and photoswitched by different laser excitations of 561, 405, and 488 nm. Fluorescence emission was detected using a SPCM-AQRH14 fiber coupled avalanche photodiode (Pacer).

### Protein crystallography

K-GECO1 DNA was cloned into pRSET-A with a short N-terminal hexahistidine purification tag (MHHHHHHGSVKLIP…, tag underlined). K-GECO1 was expressed in T7 Express *E. coli* cells (New England Biolabs) for 36 h in autoinduction medium [51] supplemented with 100 mg/L ampicillin. *E. coli* pellets were lysed in B-PER (Thermo Fisher Scientific) supplemented with 1 mg/mL lysozyme followed by sonication. Insoluble cell debris was removed from the lysate by centrifugation for 20 min at 25,000 *g*, and soluble K-GECO1 protein was purified by immobilized metal affinity chromatography with nickel-charged Profinity resin (Bio-Rad), washed with 10 mM imidazole and eluted with 100 mM imidazole in Tris-buffered saline. K-GECO1 was further purified by size exclusion chromatography using a Superdex 200 column (GE Healthcare Life Sciences) with 10 mM Tris, 100 mM NaCl, pH 8.0, as the mobile phase. Purified K-GECO was concentrated to 10 mg/mL for crystallization using centrifugal concentrators (Sartorius Vivaspin, 10,000 molecular weight cut-off (MWCO)). Purified K-GECO1 protein at 10 mg/mL in 10 mM Tris, 100 mM NaCl, pH 8.0, was mixed with an equal volume of a precipitant solution containing 100 mM BIS-TRIS, 20% w/v polyethylene glycol monomethyl ether 5000, pH 6.5, at room temperature in a sitting-drop vapor diffusion crystallization tray (Hampton Research). Crystals were cryoprotected in the precipitant solution supplemented with 25% ethylene glycol. X-ray diffraction data were collected at 100 K on beamline 8.2.1 of the Advanced Light Source. Diffraction data were processed using the HKL software package [52]. The structure was solved by molecular replacement using Phaser [53], searching first for two copies of the fluorescent protein domain fragment using a single molecule of mKate (PDB ID 3BXB) as the search model, followed by two copies each of the separated N- and C-terminal lobes of the Ca^2+^-bound calmodulin domain using fragments of PDB ID 3SG3. Iterative model building in Coot [54] and refinement in Refmac [55] produced the K-GECO1 model, with two copies of K-GECO1 in the asymmetric unit. The K-GECO1 model was deposited at the PDB with the accession code 5UKG.

### Cell culture and imaging

To characterize the K-GECO variants in HeLa cells, the cells were maintained in Dulbecco’s modified Eagle medium supplemented with 10% fetal bovine serum (FBS, Thermo Fisher Scientific), penicillin-streptomycin (Thermo Fisher Scientific), GlutaMAX (Thermo Fisher Scientific) at 37 °C with 5% CO_2_. To construct the mammalian expression plasmid, pcDNA3.1(+) and the K-GECO variant were both digested with *Xho*I and *Hind*III, and the digested plasmid backbone and insert were purified by gel electrophoresis, followed by ligation and sequencing confirmation. Transient transfections of pcDNA3.1(+)-K-GECO plasmids were performed using Lipofectamine 2000 (Thermo Fisher Scientific). HeLa cells (60–70% confluency) on 35 mm glass bottom dishes (In vitro Scientific) were transfected with 1 μg of plasmid DNA, using Lipofectamine 2000 (Thermo Fisher Scientific) according to the manufacturer’s instructions. The cells were imaged 24 h after the transfection. Immediately prior to imaging, cells were washed twice with Hanks balanced salt solution (HBSS) and then 1 mL of 20 mM HEPES buffered HBSS (HHBSS) was added. Cell imaging was performed with an inverted Eclipse Ti (Nikon). The AquaCosmos software package (Hamamatsu) was used for automated microscope and camera control. Cells were imaged with a 20× objective lens. To image the histamine-induced Ca^2+^ dynamics, cells were imaged with a 200 ms exposure acquired every 5 s for a duration of 30 min. Approximately 60 s after the start of the experiment, histamine (10 μL) was added to a final concentration of 5 mM. The oscillation was imaged for 20 min, EGTA/ionomycin (40 μL) in HHBSS was added to a final concentration of 2 mM EGTA and 5 μM of ionomycin. After 5 min, Ca^2+^/ionomycin (40 μL) in Ca^2+^ and Mg^2+^-free HHBSS was added to a final concentration of 5 mM Ca^2+^ and 5 μM of ionomycin.

To characterize K-GECO variants in cultured dissociated neurons, the procedure was done as previously reported [29]. Dissociated E18 Sprague–Dawley hippocampal cells were purchased from BrainBits LLC. The cells were grown on a 35-mm glass-bottomed dish (In Vitro Scientific) containing NbActiv4 medium (BrainBits LLC) supplemented with 2% FBS, penicillin-G potassium salt (50 units/ml), and streptomycin sulfate (50 mg/ml). Half of the culture media was replaced every 4 or 5 days. Cells were transfected on day 8 using Lipofectamine 2000 (Thermo Fisher Scientific) following the manufacturer’s instructions with the following modifications. Briefly, 1–2 μg of plasmid DNA and 4 μl of Lipofectamine 2000 (Thermo Fisher Scientific) were added to 100 μl of NbActive4 medium to make the transfection medium and incubated at room temperature for 10–15 min. Half of the culture medium (1 ml) from each neuron dish was taken out and combined with an equal volume of fresh NbActiv4 medium (supplemented with 2% FBS, penicillin-G potassium salt, and streptomycin sulfate) to make a 1:1 mixture and incubated at 37 °C and 5% CO_2_. Then, 1 ml of fresh conditioned (at 37 °C and 5% CO_2_) NbActiv4 medium was added to each neuron dish. After the addition of transfection medium, the neuron dishes were incubated for 2–3 h at 37 °C in a CO_2_ incubator. The medium was then replaced using the conditioned 1:1 mixture medium prepared previously. The cells were then incubated for 48–72 h at 37 °C in a CO_2_ incubator before imaging. Fluorescence imaging was performed in HHBSS on an inverted Nikon Eclipse Ti-E microscope equipped with a 200 W metal halide lamp (PRIOR Lumen), 60× oil objectives (numerical aperture, NA = 1.4; Nikon), a 16-bit QuantEM 512SC electron-multiplying CCD camera (Photometrics), and a TRITC/Cy3 filter set (545/30 nm excitation, 620/60 nm emission, and a 570LP dichroic mirror, Chroma). For time-lapse imaging, neurons were imaged at an imaging frequency of 100 Hz with 4 × 4 binning. For photoactivation comparison, cells expressing K-GECO1 and R-GECO1 were stimulated with pulses of blue laser light (405 nm, 5 mW/mm^2^).

To compare the K-GECO1 and red GECIs in stimulated cultured neuron cells, the procedure was done as previously reported [24]. Briefly, red GECIs were expressed after electroporation into rat primary hippocampal neurons (P0) using the Nucleofector system (Lonza). For stimulation, action potentials were evoked by field stimulation. The TxRed filter set (540–580 nm excitation, 593–668 nm emission, and 585-nm-long pass dichroic mirror) was used for illumination. Responses were quantified for each cell as the change in fluorescence divided by the baseline fluorescence before stimulation. The signal-to-noise ratio was quantified as the peak fluorescence signal over the baseline, divided by the standard deviation of the fluorescence signal before the stimulation.

iPSC-CMs were purchased from Axol Bioscience. Cells were plated in two wells of a six-well plate and cultured for 4 days in Cardiomyocyte Maintenance Medium (Axol Bioscience) to 60–80% confluency. Cells then were then transferred to fibronectin-coated (1%) coverslips and imaged in Tyrode’s buffer. Cells were transfected using transfection reagent Lipofectamine 2000 (Invitrogen). An inverted microscope (Zeiss) equipped with a NA 1.4, 63× objective lens (Zeiss) and a pE-4000 multi-wavelength LED light source (CoolLED) was used. Blue (470 nm) and green (550 nm) excitation were used to illuminate ChR2-EYFP and red GECIs, respectively. The green fluorescent protein filter set (excitation 480/10 nm, 495 nm long pass dichroic mirror, emission 525/50 nm) and the RFP filter set (excitation 545/30, 565 nm long pass dichroic mirror, emission 620/60 nm) were used to visualize ChR2-EYFP and K-GECO or R-GECO, respectively. Optical stimulation was achieved with the 470-nm LED light at a power density of 0.19 W/cm^2^ and a pulse duration of 150 ms. Fluorescence signals were recorded using an ORCA-Flash4.0LT sCMOS camera (Hamamatsu) controlled by ImageJ [56].

### Organotypic hypothalamic rat brain slice imaging

To prepare organotypic brain slices, experiments were done on neonatal rat coronal brain slices containing the VMN of the hypothalamus. In brief, postnatal 0–1-day-old Sprague–Dawley rats were anesthetized with 2–3% isoflurane until the paw reflex disappeared. Following decerebration, the brain was isolated in ice-cold divalent cation-free HBSS (Thermo Fisher Scientific) with 1 mM CaCl_2_ and 1.3 mM MgSO_4_. The brain was glued caudal side down to a metal plate and serial sections of 400 μm thickness were made using a vibratome (Leica Microsystems). Sectioning was stopped when the third ventricle became visible and two VMN-containing slices of 250 μm thickness were cut. Individual slices were placed on a sterile 0.4-μm-pore-membrane cell culture insert (Millipore). The insert and slice were then transferred to a 35-mm-diameter culture dish (Corning) containing 1.5 ml of NbActiv4 medium (BrainBits) supplemented with 5% FBS, penicillin-G potassium salt (50 units/ml), and streptomycin sulfate (50 μg/ml). Slices were cultured at 37 °C in an incubator (Thermo Fisher Scientific) under gassing with 5% CO_2_.

For transfection of organotypic slices, after 8–10 days of organotypic slice culturing, the VMN areas were transfected with an electroporation technique as previously described [47]. Specifically, the insert with the slice was placed on a platinum plate petri dish electrode (Bex Co Ltd) and electroporation buffer (HBSS with 1.5 mM MgCl_2_ and 10 mM D-glucose) was filled between the electrode and the membrane. Plasmids of pcDNA3.1-K-GECO1 were dissolved in the electroporation buffer at a concentration of 1 μg/ml and 10 μl of this solution was added to just cover the slice. Then, a square platinum electrode (Bex Co Ltd) was placed directly above the slice. Five 25-V pulses (5 ms duration and interval 1 s) were applied twice (the second time with reversed polarity) using a pulse stimulator (Sequim) and an amplifier (Agilent). The electroporation buffer was replaced with supplemented NbActiv4 medium and slices were returned to the incubator.

To image the cytosolic Ca^2+^ dynamics using K-GECO1, an upright FV1000 confocal microscope equipped with FluoView software and a 20× XLUMPlanF1 water immersion objective (NA 1.0) was used (Olympus). The Millipore insert containing a transfected brain slice was placed in a custom-made chamber and mechanically fixed with a platinum harp. The slices were then perfused at 31 °C with artificial cerebrospinal fluid containing (in mM) 120 NaCl, 3 KCl, 1 CaCl_2_, 1.3 MgSO_4_, 26 NaHCO_3_, 1.25 NaH_2_PO_4_, and 10 D-glucose (the pH was adjusted to 7.4 by gassing with 95% O_2_ plus 5% CO_2_), at a flow rate of 5 ml/min using a peristaltic pump (Watson-Marlow). For single-color confocal Ca_i_ imaging, K-GECO-transfected VMN neurons were exposed to excitation with 543-nm laser light and emissions were collected from 560 to 660 nm using a variable barrier filter. Images were acquired at × 1–3 digital zoom at a frame resolution of 512 × 512 and with a 2 μs/pixel scanning rate resulting in image acquisition at 1.12 frames/s. To monitor the drug-evoked cytosolic Ca^2+^ rises approximately 60 s after the start of image acquisition, 100 μM ATP (Sigma-Aldrich) was added to the artificial cerebrospinal fluid for 90 s. To compare the K-GECO1 signal with that of a chemical Ca^2+^ fluorescent dye, transfected slices were stained with the membrane-permeant (AM) variant of green Fluo-4 by focal application. In brief, 0.5 mM of Fluo-4-AM was filled into a broken patch pipette with an outer diameter of ~10 μm and subsequently pressure-injected (25–50 mmHg) for 10 min [57, 58] at 30–50 μm depth into the slice in the vicinity of the K-GECO1-transfected VMN neurons. This led to the uniform staining of cells in a radius of 150–200 μm from the injection site. For dual-color imaging of K-GECO1- and Fluo-4-based Ca^2+^ responses, double-labeled neurons were excited with a 488-nm laser and emissions were simultaneously collected in two channels from 500 to 520 nm for Fluo-4 and 570 to 670 nm for K-GECO1 using variable barrier filters.

### Imaging of zebrafish spinal sensory neurons

Mitfa^w2/w2^ roy^a9/a9^ (Casper) zebrafish were maintained under standard conditions at 28 °C and a 14:10 hr light:dark cycle. Embryos (cell stage 1–2) of Tg (elavl3:GAL4-VP16) [59] were injected with 25 ng/μl DNA plasmids encoding the K-GECO variants under the control of the 10xUAS promoter, and 25 ng/μL Tol2 transposase mRNA diluted in E3 medium. Three-day post-fertilization embryos showing expression in spinal sensory neurons (RB cells) were paralyzed by a 5-min bath application of 1 mg/ml a-bungarotoxin (Sigma, 203980). Larvae were mounted on their side in a field stimulation chamber (Warner, RC-27NE2) with 1.5% low-melting-point agarose and imaged using a custom-built two-photon microscope equipped with a resonant scanner. The light source was an Insight DS Dual femtosecond laser (Spectra-Physics) running at 1140 nm. The objective was a 25× 0.95 NA water immersion lens (Leica). Functional images (512 × 256 pixels) were acquired using ScanImage 5 (vidriotechnologies.com) at 7.5 Hz. The approximate laser power at the sample was measured using a slide power meter (Thorlabs) and 3 and 20 mW were used for functional imaging. Trains of 1, 2, 5, 10, and 20 field stimuli (1 ms pulse width at 50 Hz) were applied with a stimulator (NPI ISO-STIM). The stimulation voltage was calibrated to elicit an identifiable response to a single pulse in RB cells without stimulating muscle cells. Regions of interest (ROIs) were selected manually, and data were analyzed using MATLAB (MathWorks).

### Mouse V1 imaging

For in vivo mouse V1 imaging, the procedure was done as previously reported [24]. Briefly, AAV injection was used for expression of K-GECO1 in mouse V1 neurons. After injection of the virus, a cranial window was implanted. The animal was then placed under a microscope at 37 °C and anesthetized during imaging. A custom-built two-photon microscope was used for imaging with a 1100-nm pulse laser as light source and a 16× 0.8 NA water immersion lens as objective. The laser power was 100–150 mW at the front aperture of the objective lens. The moving grating stimulus trial consisted of a blank period followed by a drifting sinusoidal grating with eight drifting directions with 45° separation. The gratings were presented with an LCD screen placed in front of the center of the right eye of the mouse. For the fixed tissue analysis, the mice were anesthetized and transcardially perfused. The brains were then removed and post-fixed. Sections of the brains were coverslipped and imaged using confocal microscopy (LSM 710, Zeiss).

### Statistical analysis

All data are expressed as means ± standard deviation. Sample sizes (*n*) are listed for each experiment. For V1 functional imaging, the ANOVA test (*p* = 0.01) was used to identify responsive cells for each of the grating stimuli.

## Additional files

Additional file 1: Figure S1. Protein sequence alignment of K-GECO1, R-CaMP2, R-GECO1, and RCaMP1h. Reserved residues are colored in blue. Different residues are highlighted in red. Structural information is indicated with colored bars below the aligned sequences. (TIF 675 kb)

Additional file 2: Table S1. In vitro photophysical characteristics of K-GECO1, R-GECO1, and RCaMP1h (-/+ Ca^2+^) (DOC 34 kb)

Additional file 3: Figure S2. In vitro photoactivation characterization of K-GECO1, R-GECO1, and RCaMP1h. **a** Representative K-GECO1 fluorescence response to switching between 4 s of illumination with a 561-nm (6.13 W/cm^2^) laser and 1 s with a 405- nm (1.76 W/cm^2^) or 488-nm (6.13 W/cm^2^) laser in the presence and absence (EGTA buffer) of Ca^2+^. **b** Representative R-GECO1 fluorescence response with switching between 4 s of a 561-nm (3.83 W/cm^2^) laser and 1 s of a 405-nm (0.08 W/cm^2^) or 488-nm (3.83 W/cm^2^) laser in both Ca^2+^ buffer and Ca^2+^-free buffer. **c** Representative RCaMP1h fluorescence response with switching between 4 s of a 561-nm (3.83 W/cm^2^) laser and 1 s of a 405-nm (0.08 W/cm^2^) or a 488-nm (3.83 W/cm^2^) laser in both Ca^2+^ buffer and Ca^2+^-free buffer. **d** Percentage fluorescence change of K-GECO1, R-GECO1, and RCaMP1h in Ca^2+^-free buffer after applying 1 s of a 488-nm laser with various intensities when illuminated with a 561-nm laser (*n* = 5 photoswitching cycles for K-GECO1; *n* = 6–9 photoswitching cycles for R-GECO1; *n* = 6 photoswitching cycles for RCaMP1h). Supporting numeric data are provided in Additional file 13. (TIF 1097 kb)

Additional file 4: Table S2. X-ray diffraction data collection and model refinement statistics. (DOC 48 kb)

Additional file 5: Figure S3. Fluorescence localization and photoactivation of K-GECO1 and R-GECO1 in cultured neurons. **a** Representative fluorescence image of a K-GECO1-transfected cultured hippocampal neuron. **b** Representative fluorescence image of a R-GECO1-transfected cultured hippocampal neuron. Fluorescent puncta structures are indicated by the arrowhead. **c** K-GECO1 fluorescence response in neurons when applying 405-nm laser illumination. **d** R-GECO1 fluorescence response in neurons when applying 405-nm laser illumination. Supporting numeric data are provided in Additional file 14. (TIF 480 kb)

Additional file 6: Figure S4. K-GECO1 expression patterns in zebrafish Rohon–Beard (RB) cells. **a** Schematic view of the image window. **b** Representative images of K-GECO1 expression in RB cells. **c** Representative images of jRGECO1a (with NES) expression in RB cells. (TIF 1782 kb)

Additional file 7: Figure S5. K-GECO1 expression patterns in mouse V1 neurons. **a** Representative images of K-GECO1 (with NES) expression in a fixed tissue section from a mouse V1. **b** Representative image and zoom-in view of K-GECO1 expression in functional imaging of mouse V1 neurons. (TIF 2598 kb)

Additional file 8: Numeric data for Fig. 2. (XLSX 25 kb)

Additional file 9: Numeric data for Fig. 3a,b. (XLSX 86 kb)

Additional file 10: Numeric data for Fig. 4. (XLSX 123 kb)

Additional file 11: Numeric data for Fig. 5. (XLSX 101 kb)

Additional file 12: Numeric data for Fig. 6e–h. (XLSX 13 kb)

Additional file 13: Numeric data for Additional file 3: Figure S2. (XLSX 259 kb)

Additional file 14: Numeric data for Additional file 5: Figure S3. (XLSX 88 kb)
