# Correction to: A genetically encoded Ca^2+^ indicator based on circularly permutated sea anemone red fluorescent protein eqFP578

**DOI:** 10.1186/s12915-019-0707-8

**Published:** 2019-10-30

**Authors:** Yi Shen, Hod Dana, Ahmed S. Abdelfattah, Ronak Patel, Jamien Shea, Rosana S. Molina, Bijal Rawal, Vladimir Rancic, Yu-Fen Chang, Lanshi Wu, Yingche Chen, Yong Qian, Matthew D. Wiens, Nathan Hambleton, Klaus Ballanyi, Thomas E. Hughes, Mikhail Drobizhev, Douglas S. Kim, Minoru Koyama, Eric R. Schreiter, Robert E. Campbell

**Affiliations:** 1 Department of Chemistry, University of Alberta, Edmonton, Alberta T6G 2G2 Canada; 2 Janelia Research Campus, Howard Hughes Medical Institute, Ashburn, Virginia 20147 USA; 3 Department of Cell Biology and Neuroscience, Montana State University, Bozeman, MT 59717 USA; 4 Department of Physiology, University of Alberta, Edmonton, Alberta T6G 2H7 Canada; 5 LumiSTAR Biotechnology Incorporation, Nangang Dist, Taipei City, 115 Taiwan

**Correction to: BMC Biol (2018) 16(1):9**

**https://doi.org/10.1186/s12915-018-0480-0**

In the online version of the article [1], Figure S1 was mistakenly replaced with Figure 1.

Please see the correct Additional file 1: Figure S1 below.

The publisher apologizes for any inconvenience caused to the authors and the readers.

## Supplementary information

**Additional file 1:** **Figure S1.** Protein sequence alignment of K-GECO1, R-CaMP2, R-GECO1, and RCaMP1h. Conserved residues are colored in blue. Different residues are highlighted in red. Structural information is indicated with colored bars below the aligned sequences.
