# Supplementary figures and images for: A genetically encoded Ca2+ indicator based on circularly permutated sea anemone red fluorescent protein eqFP578
Source: BMC Biol. 2018 Jan 16;16:9. doi: 10.1186/s12915-018-0480-0 (PMC5771076; doi:10.1186/s12915-018-0480-0)

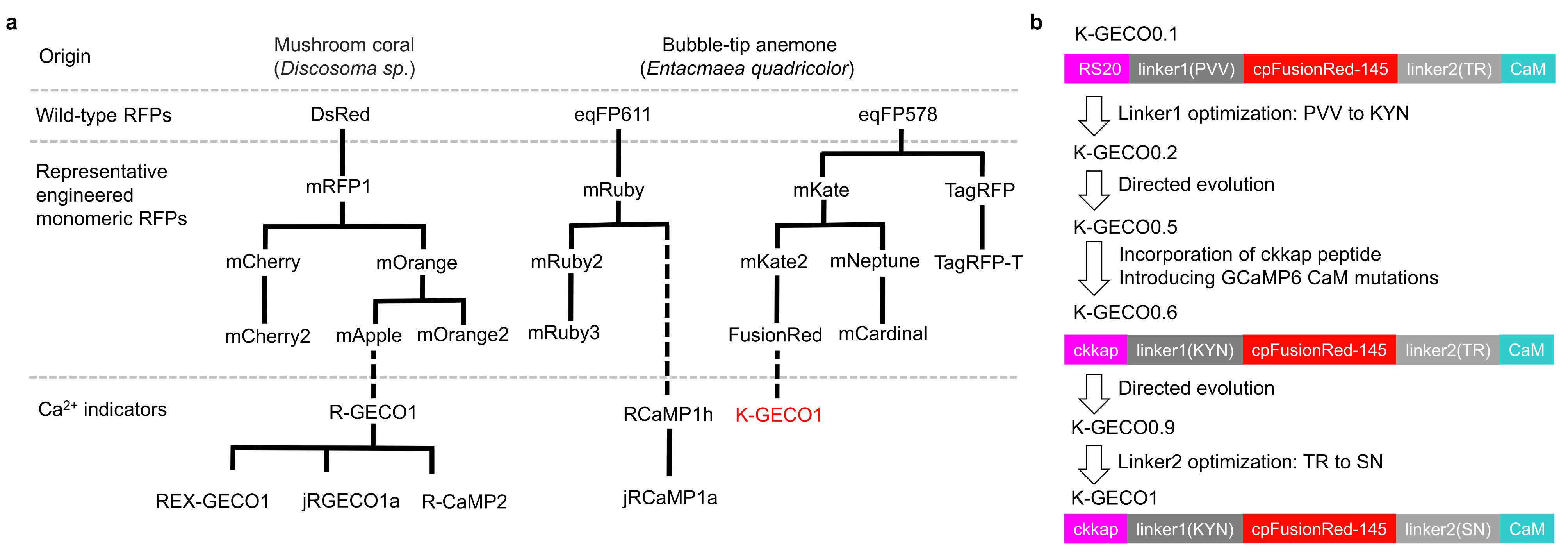

Supplement: Supplementary file 1 — Protein sequence alignment of K-GECO1, R-CaMP2, R-GECO1, and RCaMP1h. Reserved residues are colored in blue. Different residues are highlighted in red. Structural information is indicated with colored bars below the aligned sequences. (TIF 675 kb) [file 12915_2018_480_MOESM1_ESM.tif]

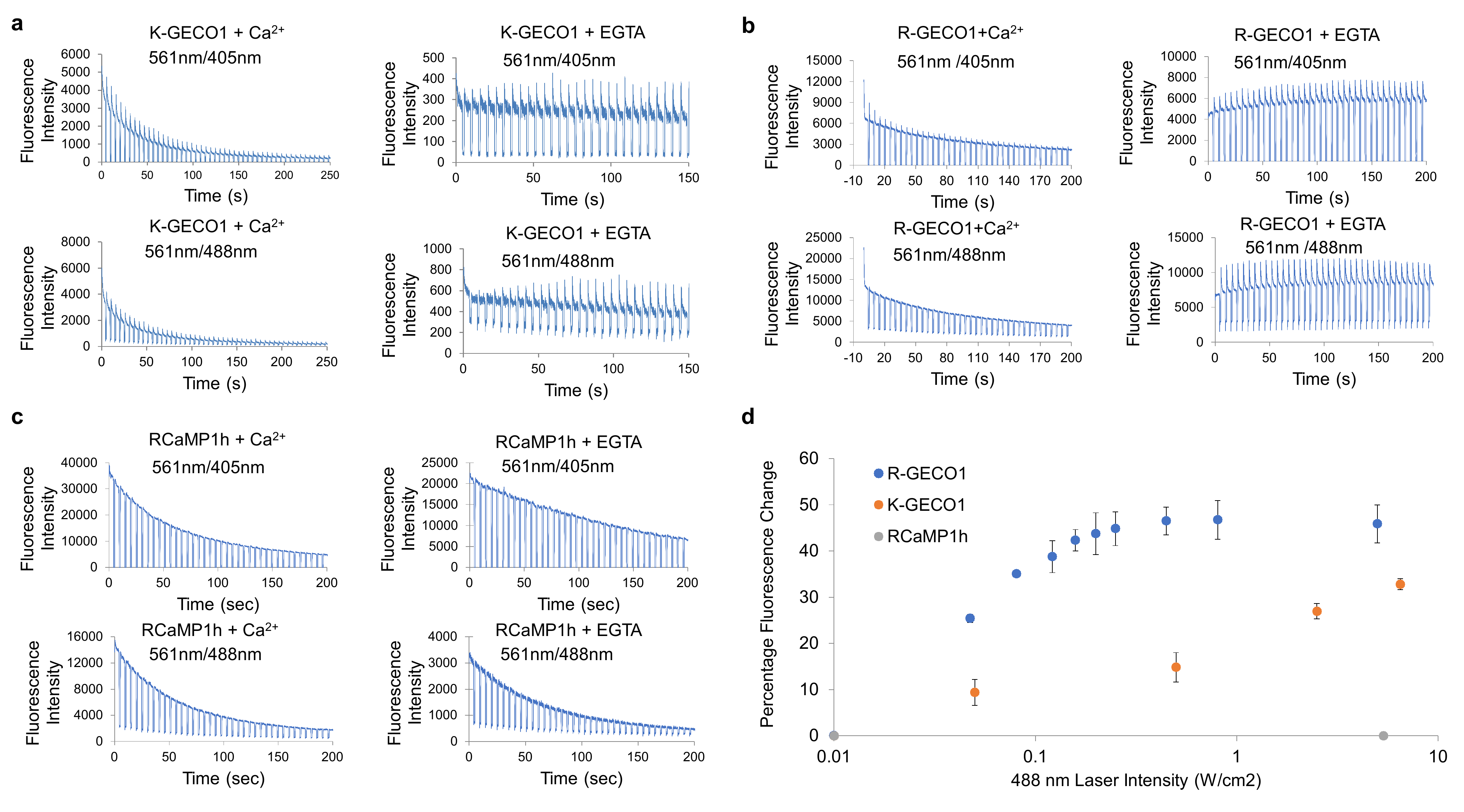

Supplement: Supplementary file 3 — In vitro photoactivation characterization of K-GECO1, R-GECO1, and RCaMP1h. a Representative K-GECO1 fluorescence response to switching between 4 s of illumination with a 561-nm (6.13 W/cm2) laser and 1 s with a 405- nm (1.76 W/cm2) or 488-nm (6.13 W/cm2) laser in the presence and absence (EGTA buffer) of Ca2+. b Representative R-GECO1 fluorescence response with switching between 4 s of a 561-nm (3.83 W/cm2) laser and 1 s of a 405-nm (0.08 W/cm2) or 488-nm (3.83 W/cm2) laser in both Ca2+ buffer and Ca2+-free buffer. c Representative RCaMP1h fluorescence response with switching between 4 s of a 561-nm (3.83 W/cm2) laser and 1 s of a 405-nm (0.08 W/cm2) or a 488-nm (3.83 W/cm2) laser in both Ca2+ buffer and Ca2+-free buffer. d Percentage fluorescence change of K-GECO1, R-GECO1, and RCaMP1h in Ca2+-free buffer after applying 1 s of a 488-nm laser with various intensities when illuminated with a 561-nm laser (n = 5 photoswitching cycles for K-GECO1; n = 6–9 photoswitching cycles for R-GECO1; n = 6 photoswitching cycles for RCaMP1h). Supporting numeric data are provided in Additional file 13. (TIF 1097 kb) [file 12915_2018_480_MOESM3_ESM.tif]

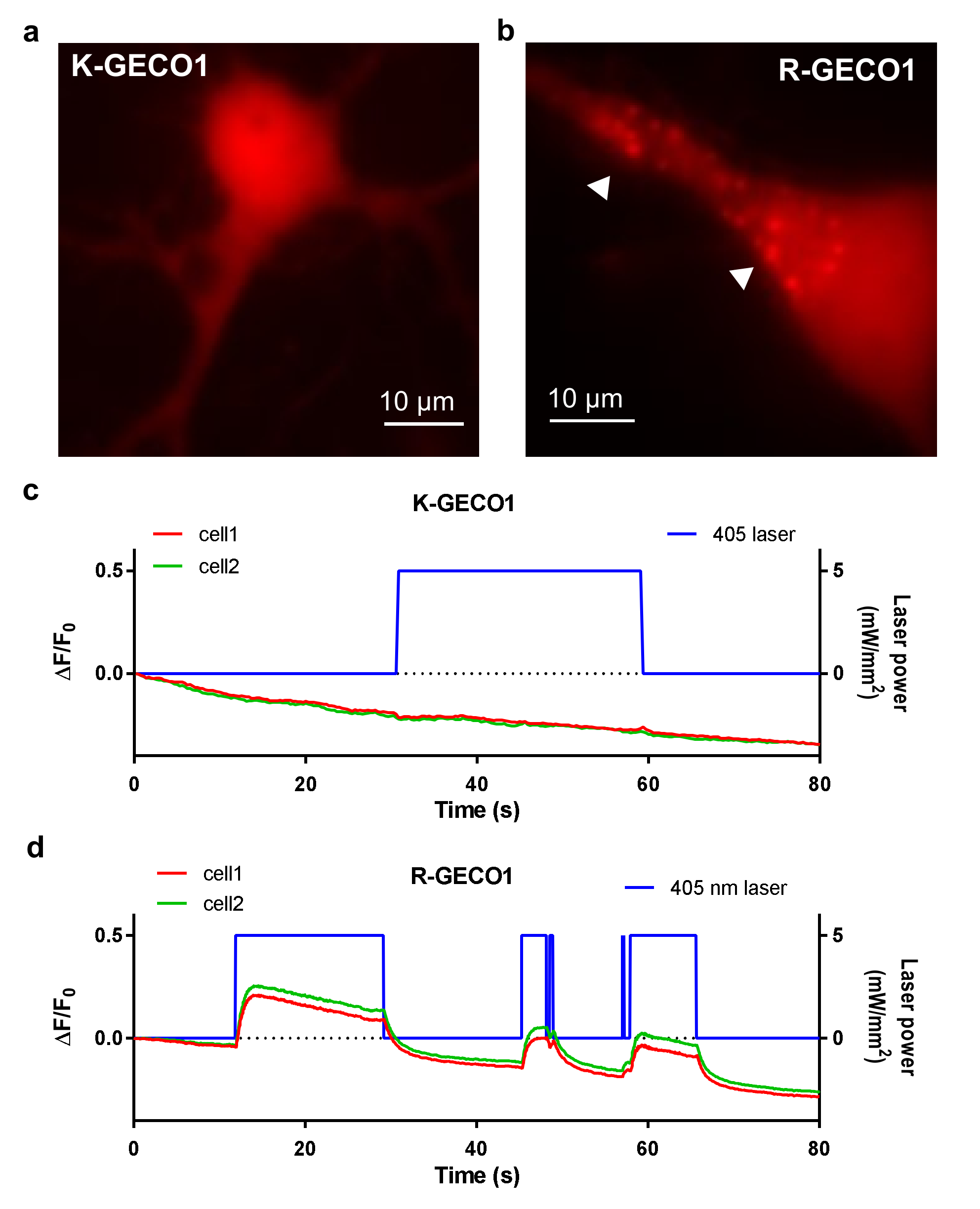

Supplement: Supplementary file 5 — Fluorescence localization and photoactivation of K-GECO1 and R-GECO1 in cultured neurons. a Representative fluorescence image of a K-GECO1-transfected cultured hippocampal neuron. b Representative fluorescence image of a R-GECO1-transfected cultured hippocampal neuron. Fluorescent puncta structures are indicated by the arrowhead. c K-GECO1 fluorescence response in neurons when applying 405-nm laser illumination. d R-GECO1 fluorescence response in neurons when applying 405-nm laser illumination. Supporting numeric data are provided in Additional file 14. (TIF 480 kb) [file 12915_2018_480_MOESM5_ESM.tif]

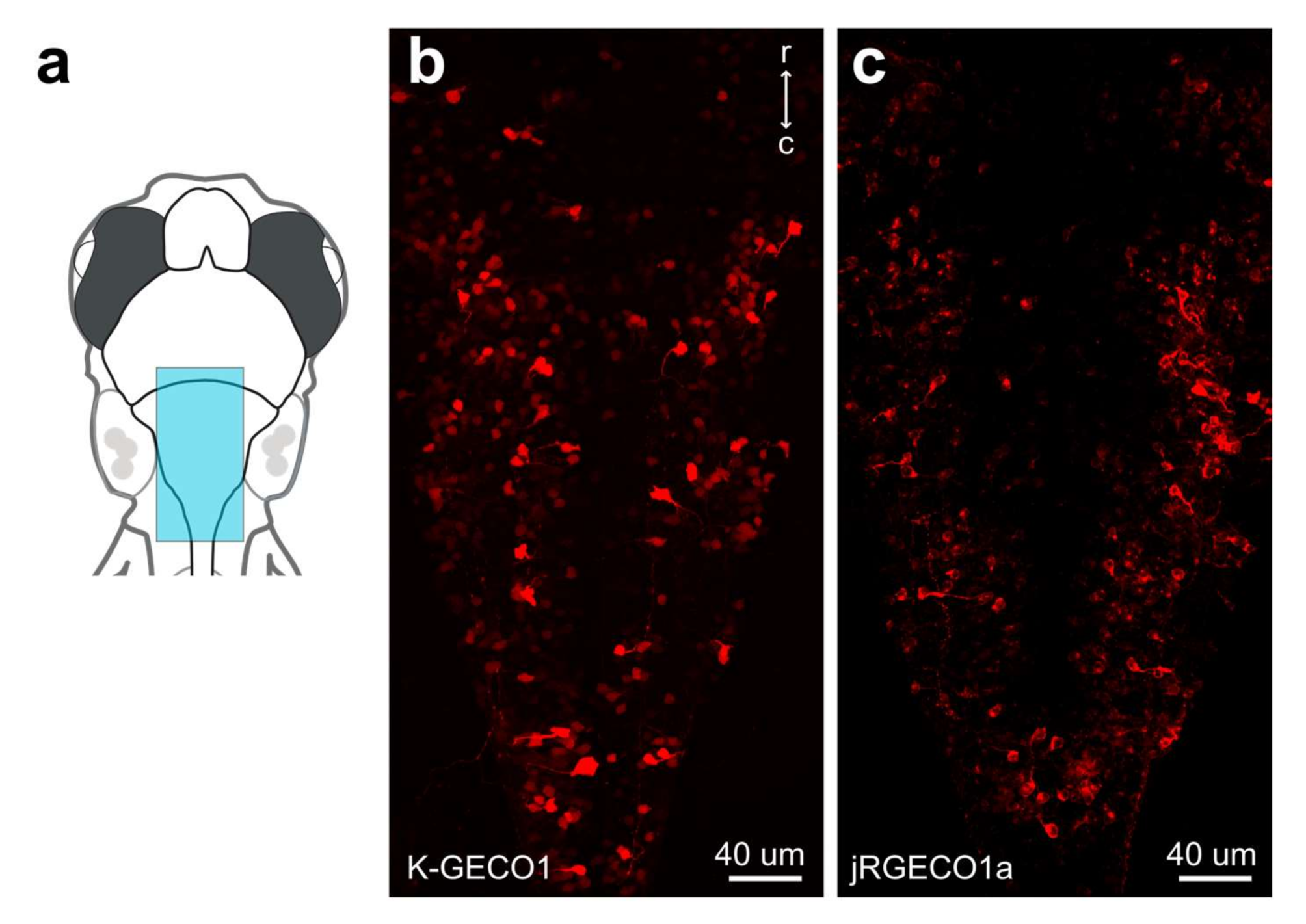

Supplement: Supplementary file 6 — K-GECO1 expression patterns in zebrafish Rohon–Beard (RB) cells. a Schematic view of the image window. b Representative images of K-GECO1 expression in RB cells. c Representative images of jRGECO1a (with NES) expression in RB cells. (TIF 1782 kb) [file 12915_2018_480_MOESM6_ESM.tif]

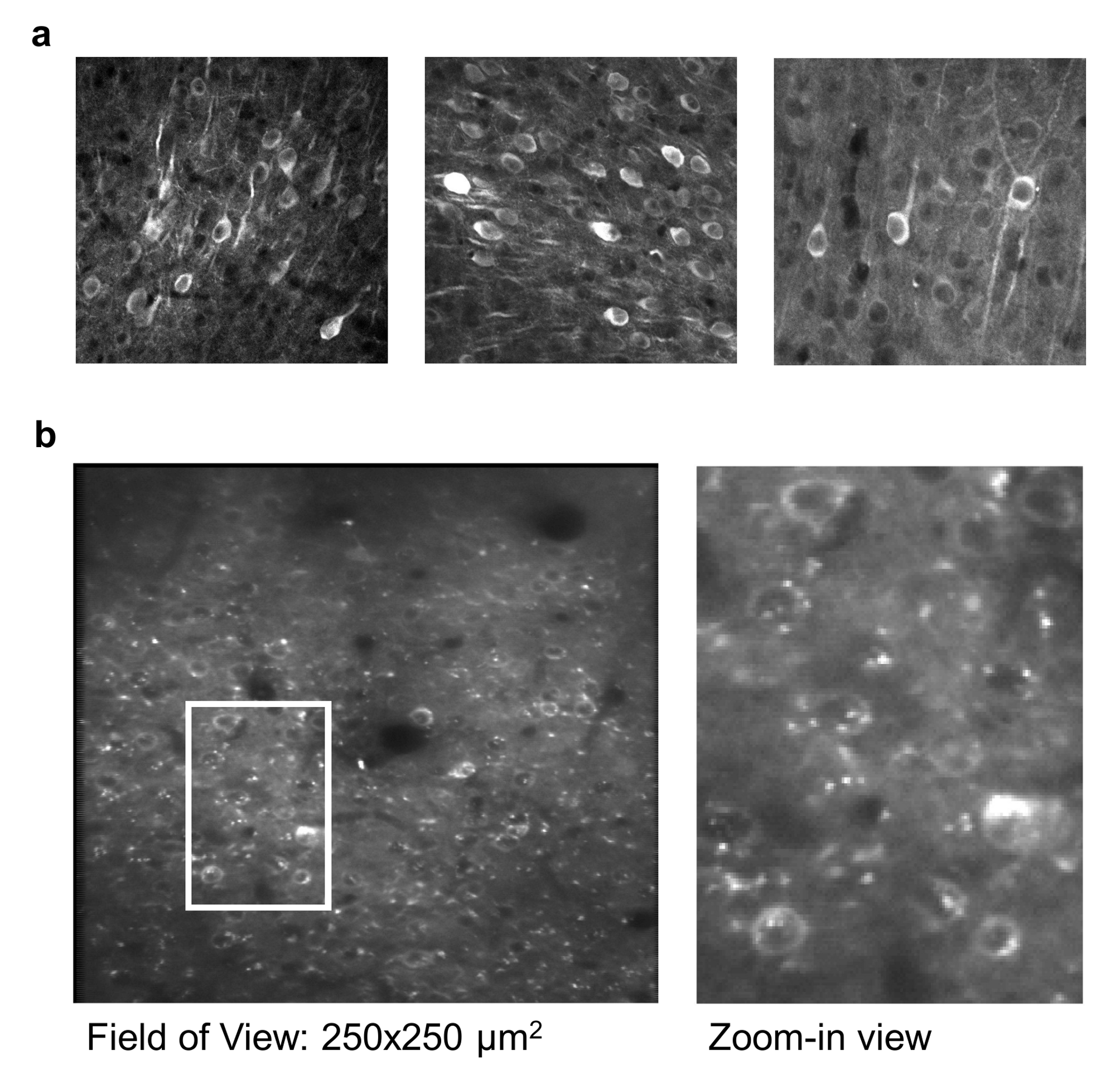

Supplement: Supplementary file 7 — K-GECO1 expression patterns in mouse V1 neurons. a Representative images of K-GECO1 (with NES) expression in a fixed tissue section from a mouse V1. b Representative image and zoom-in view of K-GECO1 expression in functional imaging of mouse V1 neurons. (TIF 2598 kb) [file 12915_2018_480_MOESM7_ESM.tif]
