# Supplementary material for: A genetically encoded Ca2+ indicator based on circularly permutated sea anemone red fluorescent protein eqFP578
Source: BMC Biol. 2018 Jan 16;16:9. doi: 10.1186/s12915-018-0480-0 (PMC5771076; doi:10.1186/s12915-018-0480-0)
Supplement: Supplementary file 2 — In vitro photophysical characteristics of K-GECO1, R-GECO1, and RCaMP1h (-/+ Ca2+) (DOC 34 kb) [file 12915_2018_480_MOESM2_ESM.doc]

**Supplementary Table 1. *In vitro* photophysical characteristics of K-GECO1, R-GECO1, and RCaMP1h (-/+ Ca^2+^).**

|  | Excitation  Maxima (nm) | Emission Maxima (nm) | Intensity fold change | Extinction Coefficient  (M^-1^cm^-1^) | Quantum Yield | *K*_d_ (nM) | Apparent  Hill Coefficient | References |
| --- | --- | --- | --- | --- | --- | --- | --- | --- |
| K-GECO1 | 568 (-) | 594 (-) | 12x | 19,000 (-) | 0.12 (-) | 165 | 1.12 | This study |
|  | 565 (+) | 590 (+) |  | 61,000 (+) | 0.45 (+) |  |  |  |
| R-GECO1 | 577 (-) | 600 (-) | 16x | 15,000 (-) | 0.06 (-) | 482 | 2.06 | 18 |
|  | 561 (+) | 589 (+) |  | 51,000 (+) | 0.20 (+) |  |  |  |
| RCaMP1h | 575 (-) | 602 (-) | 12x | 18,700 (-) | 0.14 (-) | 1300 | 2.50 | 19 |
|  | 571 (+) | 594 (+) |  | 65,100 (+) | 0.51 (+) |  |  |  |
