# Supplementary material for: A genetically encoded Ca2+ indicator based on circularly permutated sea anemone red fluorescent protein eqFP578
Source: BMC Biol. 2018 Jan 16;16:9. doi: 10.1186/s12915-018-0480-0 (PMC5771076; doi:10.1186/s12915-018-0480-0)
Supplement: Supplementary file 4 — X-ray diffraction data collection and model refinement statistics. (DOC 48 kb) [file 12915_2018_480_MOESM4_ESM.doc]

Supplementary Table 2. X-ray diffraction data collection and model refinement statistics.

|  | **K-GECO**  **PDB ID 5UKG** |
| --- | --- |
| **Data collection** |  |
| Space group | P2_1_ |
| Unit cell dimensions |  |
| a (Å) | 42.3 |
| b (Å) | 129.2 |
| c (Å) | 78.7 |
| b (deg) | 106.3 |
| Beamline | ALS 8.2.1 |
| Wavelength (Å) | 1.000 |
| Resolution range (Å) | 50 – 2.4 |
| Total reflections | 146,057 |
| Unique reflections | 30,298 |
| Completeness (%)^a^ | 94.6 (74.1) |
| I/σ^a^ | 5.1 (1.3) |
| R_sym_ (%)^a,b^ | 20.1 (58.1) |
| CC_1/2_ in highest resolution shell | 0.697 |
|  |  |
| **Refinement** |  |
| R_work_ / R_free_ (%)^c^ | 21.0/27.5 |
| Resolution range (Å) | 76 – 2.36 |
| Number of atoms (B factor) |  |
| protein | 6203 (36.9) |
| Ca^2+^ | 8 (41.8) |
| water | 64 (31.4) |
| RMSD values |  |
| Bond lengths (Å) | 0.012 |
| Bond angles (degrees) | 1.71 |
| Ramachandran (%) |  |
| Favored/Disallowed | 97.1/0.4 |
| Molprobity |  |
| Clashscore (percentile) | 2.4 (100) |
| Molprobity score (percentile) | 1.44 (99) |

^a^The number in parentheses is for the highest resolution shell.

^b^R_sym_ = Σ^i^_hkl_ |I^i^ _(hkl)_ - <I _(hkl)_>| / Σ_hkl_ <I _(hkl)_>, where I^i^ _(hkl)_ is the i^th^ measured diffraction intensity and <I _(hkl)_> is the mean of the intensity for the miller index (hkl).

^c^R_work_ = Σ_hkl_ ||F_o_ (hkl)| - |F_c_ (hkl)|| / Σ_hkl_ |F_o_ (hkl)|. R_free_ = R_work_ for 5% of reflections not included in refinement.
