# Supplementary figures and images for: Correction to: A genetically encoded Ca2+ indicator based on circularly permutated sea anemone red fluorescent protein eqFP578
Source: BMC Biol. 2019 Oct 30;17:85. doi: 10.1186/s12915-019-0707-8 (PMC6822336; doi:10.1186/s12915-019-0707-8)

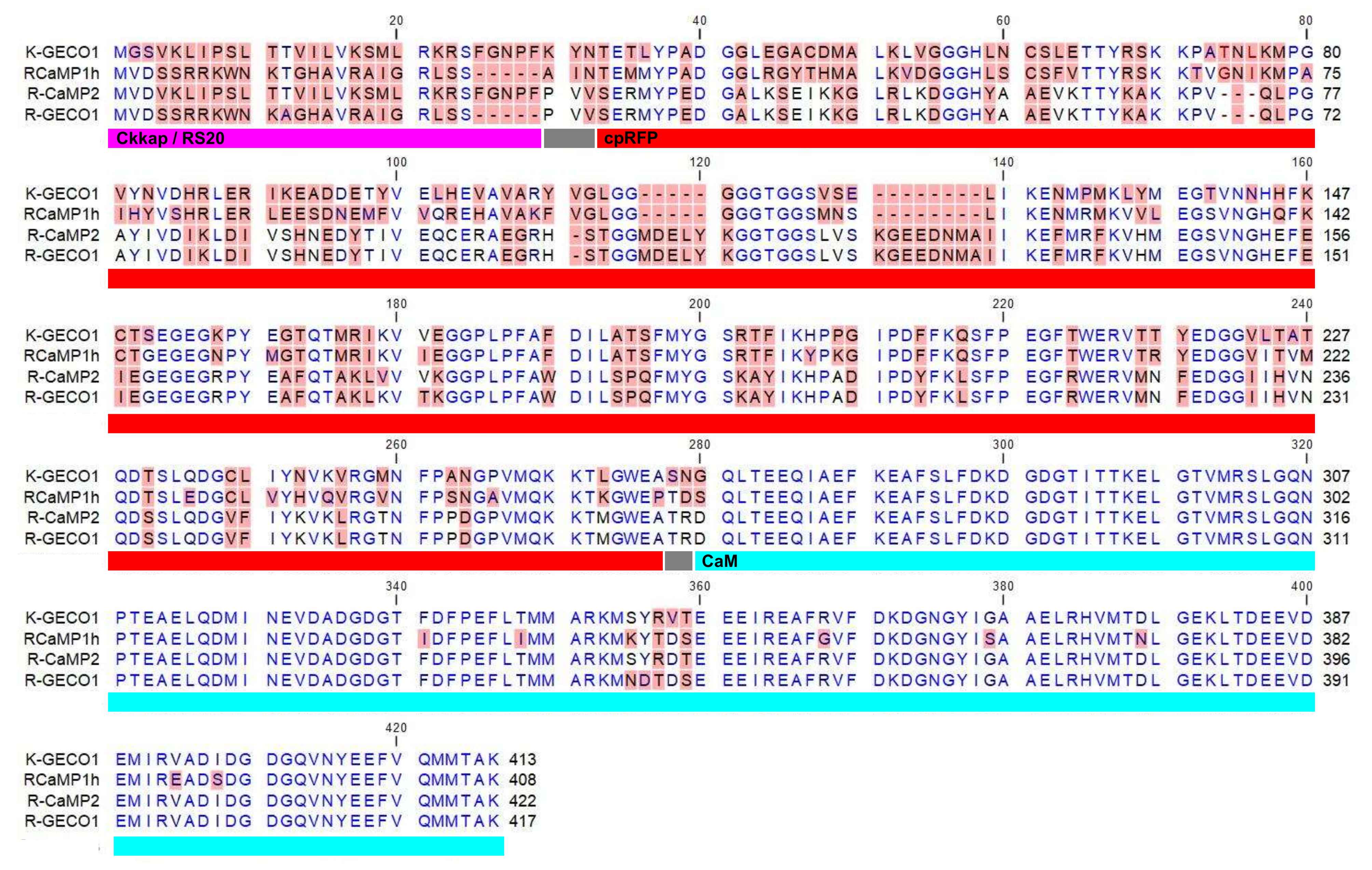

Supplement: Supplementary file 1 — Additional file 1: Figure S1. Protein sequence alignment of K-GECO1, R-CaMP2, R-GECO1, and RCaMP1h. Conserved residues are colored in blue. Different residues are highlighted in red. Structural information is indicated with colored bars below the aligned sequences. [file 12915_2019_707_MOESM1_ESM.tif]
